# Evaluation of dual-lumen pulmonary artery cannulation in extracorporeal right ventricular support

**DOI:** 10.1016/j.xjon.2026.101699

**Published:** 2026-03-04

**Authors:** Valeria Lo Coco, Michele Di Mauro, Silvia Mariani, Elham Bidar, Kasia Hryniewicz, Antonio Loforte, Thomas Fux, Sam Heuts, Dominik Wiedemann, Michal Kawcynsky, Tom Verbelen, Lars Mikael Broman, Jamila Kremer, Matteo Pozzi, Koji Takeda, Udo Boeken, Yih-Sharng Chen, Paolo Masiello, Dominik J. Vogel, Jacinta J. Maas, Andrea Ballotta, Federico Pappalardo, Sandro Gelsomino, Roberto Lorusso, PLACE Investigators, Jos Maessen, Opema Lohese, Davide Pacini, Sofia Martin Suarez, Luca Botta, Daniel Zimpfer, Anne-Kristin Schaefer, Philipp Szalkiewicz, Bart Meyns, Marie De Vos, Leen Vercaemst, Matthias Karck, Anna L. Meyer, Ana J. Holler, Matthias Jacquet-Lagrèze, Jean Francois Obadia, Jean Luc Fellahi, Amy S. Wang, Nikolaos Kalampokas, Artur Lichtenberg, Hug Aubin, Chih-Hsien Wang, Chun-Cheng Huang, Heng-Wen Chou, Severino Iesu, Generoso Mastrogiovanni, Vittoria Iennaco, Victoria M. Taylor, Nicholas A. Barret, Carlos V. Elzo Kraemer, Jorge E. Lopez Matta, Jeroen J. Janson, Camilla L'Acqua, Fabiana L. Rossi, Antonella Bertera, Giulia Maj, Andrea Audo, Stephanie Bertolin

**Affiliations:** a Cardio-Thoracic Surgery Department, Maastricht University Medical Center, Maastricht, The Netherlands; b Cardiology Department, Minneapolis Heart Institute, Minneapolis, Minn; c Cardiac Surgery Department, Sant’Orsola University Hospital, Bologna, Italy; d Molecular Medicine and Surgery Department, Karolinska Institute, Stockholm, Sweden; e Perioperative Medicine and Intensive Care Department, Karolinska University Hospital, Stockholm, Sweden; f Cardiac Surgery Department, Medical University of Vienna, Vienna, Austria; g Cardiac Surgery Department, University Hospital Leuven, Leuven, Belgium; h Intensive Care Unit, ECMO Center Karolinska, Karolinska University Hospital, Stockholm, Sweden; i Department of Physiology and Pharmacology, Karolinska Institutet, Stockholm, Sweden; j Cardiac Surgery Department, University Hospital, Heidelberg, Germany; k Cardiac Surgery Department, Louis Pradel Cardiologic Hospital, Lyon, France; l Cardiac Surgery Department, Columbia University Irving Medical Center, New York, NY; m Cardiac Surgery Department, University Hospital, Düsseldorf, Germany; n Cardiovascular Surgery Department, National University Hospital, Taipei City, Taiwan; o Emergency Cardiac Surgery - Cardio-Thoracic-Vascular Department, University Hospital San Giovanni di Dio e Ruggi D'Aragona, Salerno, Italy; p Intensive Care Medicine Department, Guy's and St Thomas' NHS Foundation Trust, London, United Kingdom; q Intensive Care Department, Leiden University Medical Center, Leiden, The Netherlands; r Intensive Care Unit Department, IRCCS Centro Cardiologico Monzino, Milano, Italy; s Cardiothoracic and Vascular Anesthesia and Intensive Care Department, SS Antonio e Biagio e Cesare Arrigo Hospital, Alessandria, Italy; t Cardiovascular Research Institute Maastricht (CARIM), Maastricht, The Netherlands

**Keywords:** dual-lumen cannulation, right ventricular failure, venovenous ECMO, hypoxemia, causal mediation

## Abstract

**Objective:**

To evaluate whether dual-lumen (DL) versus single-lumen (SL) pulmonary artery cannulation improves outcomes in patients with refractory right ventricular failure (RVF) supported with extracorporeal life support and to identify which patients benefit most.

**Methods:**

We conducted a multicenter retrospective cohort study using the international PLACE registry (2000-2020). Adults undergoing pulmonary artery cannulation for isolated RVF were included. Outcomes were in-hospital and 30-day mortality, bleeding, thromboembolic events, continuous renal-replacement therapy, and length of stay. Propensity score weighting was applied to adjust for baseline differences. Prespecified analyses tested effect modification by hypoxemia, renal function, platelet count, cannulation site, and oxygenator use. Mediation and clustering were used to explore physiological pathways and phenotypes.

**Results:**

Among 345 patients, DL cannulation was associated with lower postoperative lactate and creatinine and with fewer bleeding events. In weighted multivariable models, DL reduced the risk of bleeding and the composite of bleeding or thromboembolism (weighted odds ratio, 0.50; 95% CI 0.32-0.77; *P* = .0017 and weighted odds ratio, 0.57; 95% CI 0.39-0.84; *P* = .004). Hypoxemia significantly strengthened the survival benefit of DL, whereas cannulation site and oxygenator use did not modify outcomes. Mediation analyses indicated that the effect of DL on survival was indirect, operating through early improvement in perfusion and renal function. Unsupervised clustering identified distinct postoperative biochemical phenotypes with markedly different prognoses; DL was associated with a shift toward favorable profiles.

**Conclusions:**

In extracorporeal life support for RVF, DL pulmonary artery cannulation improves outcomes primarily by enhancing early organ function and reducing complications. Patients with baseline hypoxemia appear to benefit most, supporting a physiology-guided approach to cannulation strategy.

**Figure:**
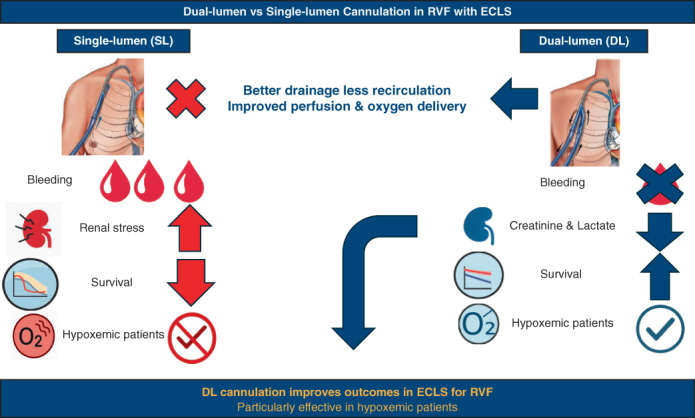
DL cannulation improves outcomes in RV failure, especially in patients with hyoxemia.

**Central Message**

Dual-lumen pulmonary artery cannulation in ECLS for RV failure improves survival by enhancing perfusion and renal function, with the greatest benefit observed in patients with hypoxemia.

**Perspective**

This study shows that dual-lumen pulmonary artery cannulation for extracorporeal right ventricular support may provide clinical benefits by improving perfusion, renal function, and reducing complications. The greatest advantage is seen in patients with hypoxemia, highlighting the value of a physiology-based, patient-tailored cannulation strategy.

Refractory acute right ventricular failure (RVF) remains a major challenge, often requiring dedicated extracorporeal life support (ECLS).^1^ Despite advances in mechanical support, the optimal ECLS mode and related cannulation strategy is debated, with potential implications for mortality and complications.^2^ A dual-lumen (DL) versus single-lumen (SL) cannula to perform dedicated ECLS for right ventricular assistance may influence outcomes through distinct physiological effects, yet previous studies are small, single-center, and largely descriptive.^1^^,^^2^

Postoperative biomarkers may clarify mechanisms of benefit, but their role in this setting remains underexplored.^3^ Advanced analytic strategies, including causal mediation and unsupervised clustering, provide opportunities to disentangle direct from indirect effects and to capture heterogeneous phenotypes.^4^^,^^5^

Leveraging data from the international retrospective PLACE, one of the largest investigations of mechanical circulatory support for RVF, this study applied inverse probability of treatment weighting (IPTW), causal mediation, structural equation modeling (SEM), and clustering. The aim of this study was to evaluate whether DL cannulation improves outcomes in patients with RVF supported with ECLS and to identify which patients benefit most from this strategy.

## Methods

### Study Design and Population

**Figure 1:**
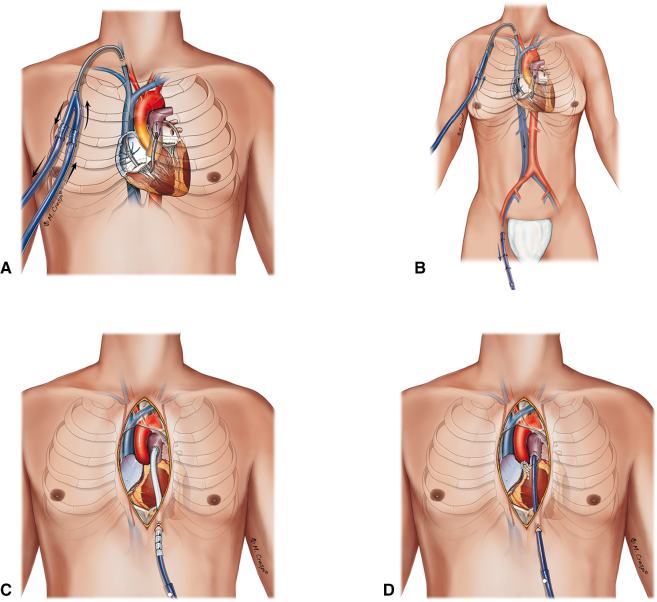
Configurations for right ventricular support. A, Percutaneous DL cannulation through the right internal jugular vein with a single cannula, draining the right atrium and returning blood to the pulmonary artery for fully percutaneous, groin-free support. B, Percutaneous SL cannulation with femoral venous drainage and return to the pulmonary artery through a surgically tunneled graft (chimney technique), typically used when jugular access is not feasible. C, Surgical SL configuration with direct cannulation of the right atrium and pulmonary artery during open chest procedures, most often in postcardiotomy right ventricular failure or when percutaneous access is contraindicated. D, Surgical DL setup with central cannulation of the right atrium and return to the pulmonary artery through a bifurcated graft system, providing controlled central support in complex operative settings. *DL*, Dual-lumen; *SL*, single-lumen.

We conducted a retrospective multicenter cohort study using the PLACE database (January 2000 to December 2020), following the STrengthening the Reporting of OBservational studies in Epidemiology guidelines^6^ (see Table E1). Adults with isolated RVF on ECLS assistance who underwent pulmonary artery cannulation with DL or SL cannula were eligible. When multiple strategies were used, the initial cannulation defined exposure. Cannulation site (peripheral through internal jugular vein vs central via sternotomy) was recorded and tested as a modifier. Oxygenator use at initiation was abstracted as binary and treated as proxy for severity. RVF was defined by American Heart Association criteria.^7^ Cannulation configurations are reported in Table E2 and Figure 1.

### Outcomes

The primary outcomes were all-cause in-hospital mortality and 30-day mortality after venovenous extracorporeal membrane oxygenation initiation. Secondary outcomes included bleeding events requiring transfusion or surgical intervention, continuous renal-replacement therapy (CRRT), thromboembolic complications, and intensive care unit/hospital length of stay. Outcome data were collected within the dedicated PLACE database and underwent validation and quality control as previously described.^8^ Definitions adhered to consensus criteria.^9^^,^^10^ The detailed distribution of outcomes by cannulation type is provided in Table E3 and illustrated in Figures E1 and E2.

### Variables and Biomarkers

Clinical and biochemical variables were selected for their relevance to ECLS for isolated RVF pathophysiology. Key biomarkers included lactate, creatinine, bilirubin, hemoglobin, and arterial oxygen tension (Pao_2_).^11^^,^^12^ Measurements were obtained pre- and postimplant to capture baseline status and early response on support.^13^ Protocols for biomarker assays were standardized and harmonized through ELSO quality control.^14^ Relevant distributions and temporal changes are shown in Figures E3 and E4. Definitions and measurement protocols for key biomarkers were adopted according to the comprehensive clinical guidance published in *Intensive Care Medicine*.^15^

### Ethics

The study used the multicenter PLACE registry of temporary isolated RV support. Protocol approval was obtained at Maastricht University Medical Center (METC 2019-1291) and at all participating centers. Written informed consent for publication of study data was waived by the Maastricht University Medical Center Medical Ethics Committee (METC 2019-1291). Where applicable, approval/waiver was obtained at each participating center, in accordance with local requirements, given the retrospective nature of the registry and the use of coded/de-identified data.

### Statistical Analysis

Missing data were handled using multiple imputation by chained equations, assuming missing at random. All variables included in the analytic models were used in the imputation process. Ten imputed datasets were generated and subsequently analyzed using Rubin's rules to pool estimates across imputations (see the Online Data Supplement). To minimize confounding inherent to observational data, we used propensity score weighting using the IPTW method to balance baseline covariates between DL and SL pulmonary artery cannula group. We considered an absolute standardized mean difference threshold of 0.2 as indicative of adequate covariate balance, in line with Austin^16^ and other literature supporting this more flexible criterion in clinical studies. Propensity scores were estimated via logistic regression incorporating clinically relevant variables, and model diagnostics, such as standardized mean differences, overlap assessments, and balance plots, were rigorously evaluated to ensure optimal covariate balance across groups.^17^ Sensitivity analyses included trimming of extreme weights and evaluation of alternative model specifications to assess robustness. Treatment effects were estimated through both univariable IPTW analyses and multivariable weighted regression models. To reduce potential bias from institutional practice patterns and temporal trends, the propensity score included center of care, year of treatment, and cannulation preference patterns across institutions. These adjustments ensured that baseline imbalances and institutional heterogeneity were appropriately accounted for. The use of complementary analytic strategies reinforced the consistency and internal validity of the findings. Subsequently, causal mediation analysis was conducted to dissect the direct and indirect pathways through which cannulation type influences clinical outcomes. This approach enabled quantification of the proportion of the total effect mediated by key postoperative biomarkers, illuminating biological mechanisms underpinning observed clinical benefits.^18^ Our use of SEM complemented these analyses by modeling complex causal relationships among variables, facilitating an integrated assessment of multiple mediators and confounders within a unified framework.^19^ Model fit was evaluated using established indices, including the root mean square error of approximation, comparative fit index, and the Tucker–Lewis index to ensure adequacy. Exploratory models evaluated cannulation site as a covariate and as an interaction term with cannula type (SL vs DL). To further characterize patient heterogeneity and identify distinct phenotypic clusters on the basis of postoperative biomarker profiles, we performed k-means clustering analysis. The optimal number of clusters was selected on the basis of within-cluster sum of squares and silhouette metrics, enabling stratification of patients into biologically and clinically meaningful subgroups.^20^ This stratification provided insights into differential risk profiles and potential therapeutic targets. All analyses were performed in R (version 3.11) using packages such as MatchIt for propensity score estimation, mediation for causal mediation, lavaan for SEM, and factoextra for clustering diagnostics. Detailed statistical methods, model specifications, and R code are provided in the Online Data Supplement. The dataset supporting the conclusions of this article is available from the corresponding author upon reasonable request and with appropriate institutional approval.

## Results

### Baseline Characteristics

We analyzed 345 patients who underwent temporary ECLS-based isolated right ventricular support. Unweighted baseline demographic and clinical characteristics are reported in Table E4, implant-related features in Table E5, and device-related complications in Table E6. Unweighted in-hospital outcomes stratified by cannulation type are shown in Table E7.

### Propensity Score Estimation and Covariate Balance

**Figure 2:**
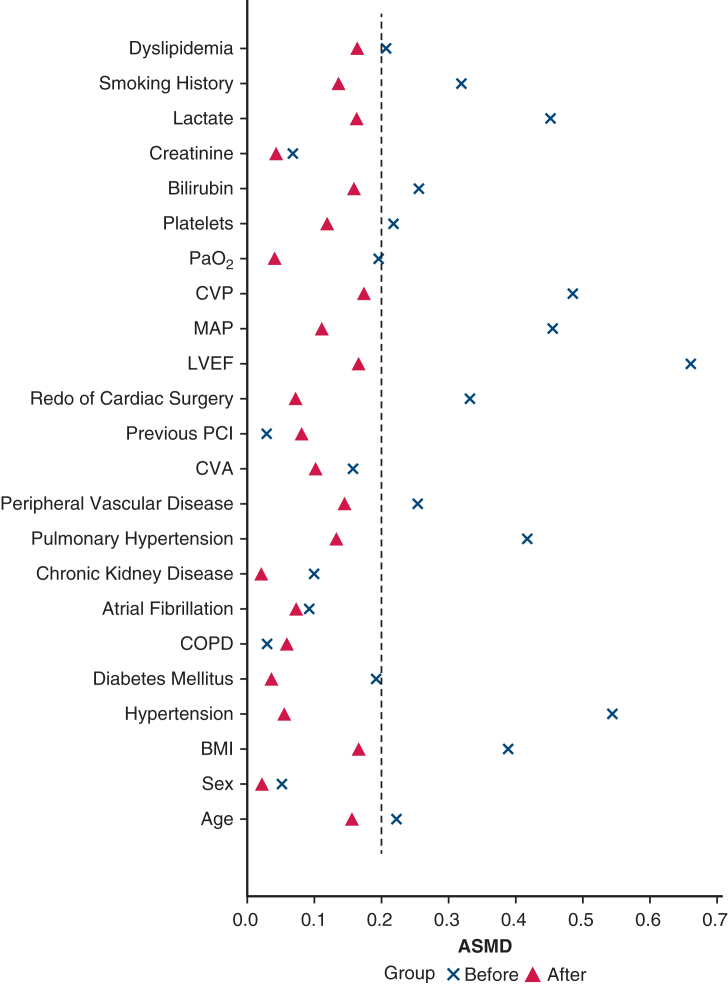
ASMD before and after weighting. Love plot of baseline covariates before and after propensity score weighting using stabilized inverse probability of treatment weights. Each *point* represents the ASMD between SL and DL groups. The *dashed line* at 0.2 marks the threshold for negligible imbalance. Covariates include demographic, clinical, and laboratory variables. Weighting reduced imbalance across most covariates, confirming effective adjustment. *ASMD*, Absolute standardized mean difference; *SL*, single-lumen; *DL*, dual-lumen; *Pa**o*_*2*_, arterial oxygen tension; *CVP*, central venous pressure; *MAP*, mean arterial pressure; *LVEF*, left ventricular ejection fraction; *PCI*, percutaneous coronary intervention; *CVA*, cerebrovascular accident; *COPD*, chronic obstructive pulmonary disease; *BMI*, body mass index.

The IPTW model substantially improved covariate balance across 23 preimplant variables, with all standardized mean differences below 0.2 (Figure 2). Additional diagnostics—including overlap plots, stabilized weight distributions, and effective sample size—are presented in Figures E5-E7. Covariate-specific balance metrics are provided in Table E8.

### Treatment Effect Estimates

**Figure 3:**
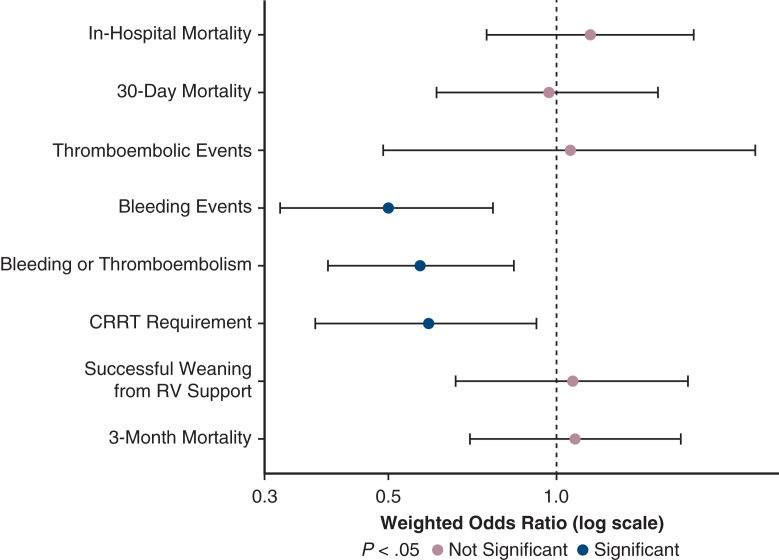
Adjusted IPTW-weighted effect estimates for primary and secondary outcomes. Results of multivariable regression models weighted by inverse probability of treatment, displaying adjusted odds ratios and adjusted mean differences with 95% CIs. DL cannulation was associated with lower risk of bleeding, composite bleeding or thromboembolism, and need for CRRT. No significant differences were observed for mortality or for hospital and intensive care unit length of stay. Outcomes are ordered as in Table E10. *IPTW*, Inverse probability of treatment weighting; *DL*, dual-lumen; *Δ Mean*, adjusted mean difference; *CRRT*, continuous renal-replacement therapy; *RV*, right ventricular.

Univariable IPTW models showed no significant mortality differences between DL and SL groups. Some differences emerged in secondary outcomes, including CRRT use and bleeding (Table E9). Multivariable IPTW-weighted logistic regression results are summarized in Table E10 and visualized in Figure 3. In these models, DL cannulation was associated with a significantly lower incidence of both bleeding events and the composite of bleeding or thromboembolism (weighted odds ratio, 0.50; 95% CI, 0.32-0.77, *P* = .0017; weighted odds ratio, 0.57; 95% CI, 0.39-0.84, *P* = .004). Descriptive temporal and institutional patterns are shown in Figure E8. Model performance was good, with discrimination area under the curve 0.76 to 0.88 and calibration Brier scores 0.114 to 0.194 (Figures E9 and 10).

### Interaction Analysis

Interaction analyses explored effect modification by baseline characteristics. Pao_2_ significantly interacted with treatment group for both in-hospital and 30-day mortality, indicating greater DL benefit in patients with hypoxemia (*P* for interaction .007 and .015, respectively). Creatinine ≥1.5 mg/dL modified the effect of DL on CRRT requirement. No interaction was found for platelet count and bleeding risk. Cannulation site (central vs peripheral) was not associated with outcomes and did not modify DL versus SL across end points (all interaction tests nonsignificant). Oxygenator use (so-called OxyRVAD mode) was not independently associated with adverse outcomes and did not interact with DL versus SL (all *P* for interaction >.05). Detailed estimates are presented in Table E11 and Figure E11.

### Mediation Analysis

**Figure 4:**
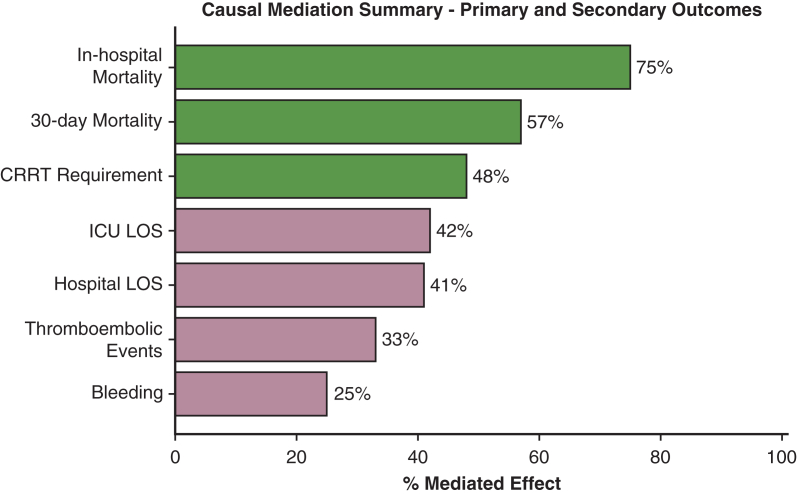
Causal mediation summary of primary and secondary outcomes. Proportion of treatment effect explained by postoperative biomarkers. Outcomes are displayed on the vertical axis, and the percentage of the total effect mediated is shown on the horizontal axis. Bars are *green* for statistically significant mediation and *mauve* for nonsignificant mediation, with numerical labels indicating the exact proportion. Only clinically plausible mediators, including postoperative lactate and creatinine, were considered. Both primary outcomes (mortality) and secondary outcomes (CRRT requirement, ICU LOS) are included, highlighting the contribution of renal dysfunction and tissue hypoperfusion. *CRRT*, Continuous renal-replacement therapy; *ICU*, intensive care unit; *LOS*, length of stay.

Causal mediation demonstrated that the association between DL and survival was not explained by a direct effect on mortality but by indirect pathways through early postoperative organ function. Postoperative lactate and creatinine mediated substantial shares of the treatment effect (32%-48%). Full numeric estimates, including nonsignificant mediators, are reported in Table E12. Representative mediation pathways are illustrated in Table 1 and Figure 4.

**Table 1:** Mediation analysis for primary and secondary outcomes

Outcome	Mediator	ACME (95% CI)	ADE (95% CI)	% Mediated	*P* value	Interpretation
30-d mortality	Creatinine	0.12 (0.03-0.21)	0.09 (−0.04 to 0.21)	57	**.004**	Significant mediation via renal function
In-hospital mortality	Lactate	0.09 (0.02-0.16)	0.03 (−0.08 to 0.13)	75	**.01**	Indirect effect through tissue hypoperfusion
CRRT requirement	Creatinine	0.14 (0.05-0.24)	0.07 (−0.06 to 0.21)	48	**.002**	Strong mediation by postimplant renal function
Bleeding	Platelets	0.04 (−0.02 to 0.11)	0.08 (−0.05 to 0.21)	25	.19	No significant mediation
Thromboembolic events	Creatinine	0.03 (−0.01 to 0.08)	0.06 (−0.07 to 0.20)	33	.26	Nonsignificant mediation effect
ICU length of stay	Lactate	0.10 (−0.03 to 0.22)	0.14 (−0.11 to 0.37)	42	.17	Potential partial mediation, NS
Hospital length of stay	Creatinine	0.07 (−0.05 to 0.18)	0.10 (−0.12 to 0.29)	41	.23	No significant indirect effect

Significant values are highlighted in bold.

ACME is the portion of the treatment effect of dual-lumen versus single-lumen cannulation that operates through a mediator such as postoperative creatinine or lactate. ADE is the remaining direct effect not explained by the mediator. “% Mediated” indicates the share of the total effect attributable to the mediator. Strong or significant mediation reflects robust evidence of an indirect pathway, whereas indirect or nonsignificant results suggest little or no mediating role. *ACME*, Average causal mediation effect; *ADE*, average direct effect; *CRRT*, continuous renal-replacement therapy; *ICU*, intensive care unit; *NS*, not significant.

### Structural Equation Modeling

**Figure 5:**
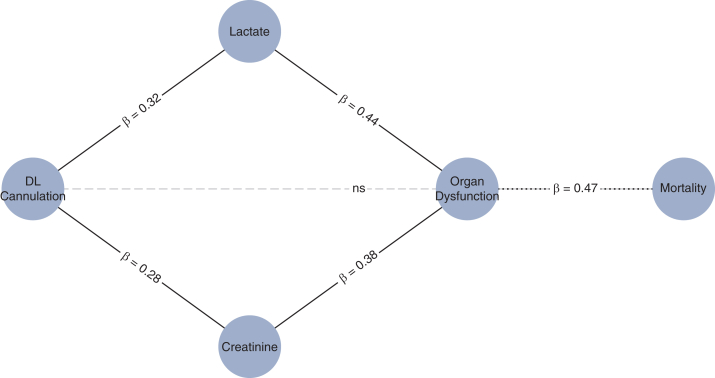
Structural equation model linking DL cannulation to reduced mortality via organ function. DL cannulation was associated with lower postoperative lactate and creatinine levels, reflecting improved perfusion and renal function. These changes reduced early organ dysfunction, which was strongly linked to lower mortality. The direct effect of cannulation on mortality was not statistically significant, indicating that the benefit was mediated through physiological improvements rather than direct hemodynamic effects. *Arrows* represent standardized path coefficients, with *solid lines* denoting significant associations and the *dashed line* a nonsignificant direct path. *DL*, Dual-lumen; *β*, standardized path coefficient.

SEM extended these findings in a unified framework. DL cannulation was associated with reductions in postoperative lactate (β = 0.32, *P* = .002) and creatinine (β = 0.28, *P* = .005), which in turn were strongly linked to mortality (β = 0.44 and β = 0.38, both *P* < .005). The direct path from DL to mortality was not significant (β = 0.12, *P* = .22). Model fit was excellent (comparative fit index = 0.97, root mean square error of approximation = 0.042, standardized root mean squared residual = 0.038). The full SEM diagram is presented in Figure 5, with additional specifications shown in Figure E12.

### Nonlethal Complications

Analysis of complications indicated indirect effects of DL on thromboembolism via renal stress. Figure E13 demonstrates a significant indirect path from DL through creatinine to thromboembolism. No significant mediation was observed for platelet count and bleeding. Figure E14 provides an interaction plot of DL and baseline platelet count on bleeding risk, showing no meaningful effect modification.

### Phenotypic Stratification

**Figure 6:**
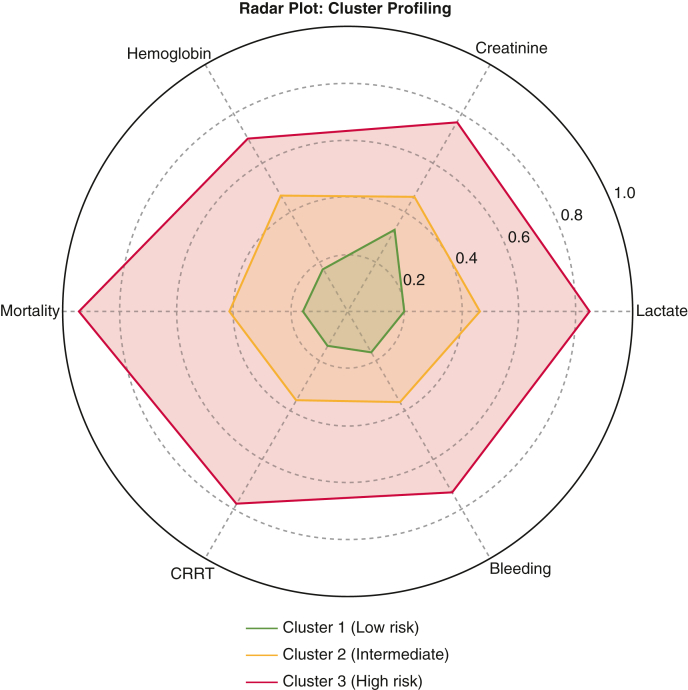
Radar plot depicting clinical profiling of patient clusters. Comparison of 3 patient clusters identified through unsupervised analysis. Cluster 1 (*green*) corresponds to low risk, Cluster 2 (*yellow*) to intermediate risk, and Cluster 3 (*red*) to high risk. Greater lactate, creatinine, mortality, CRRT, and bleeding indicate worse clinical status, whereas greater hemoglobin is associated with lower risk. The chart provides a visual overview of the distinct profiles and relative risk across the 3 groups. *CRRT*, Continuous renal-replacement therapy.

Unsupervised k-means clustering (k = 3) identified clinically distinct postoperative phenotypes (Figure 6). Cluster 1 (low lactate, normal creatinine, high hemoglobin) had favorable prognosis (hospital mortality 9.4%), Cluster 3 (high lactate, high creatinine, low hemoglobin) had poor outcomes (mortality 56.7%), and Cluster 2 showed intermediate profiles. Differences in mortality were highly significant (*P* < .001). Cluster membership was also associated with CRRT (*P* < .001), bleeding (*P* = .02), and length of stay (*P* < .01). Outcomes across clusters are detailed in Table E13, with visualizations in Figures E15.

## Discussion

In acute RVF treated with dedicated ECLS, cannulation strategy is often considered a technical choice on the basis of anatomy or operator preference. Our findings indicate instead that the choice between DL and SL cannulation has direct clinical implications. DL cannulation was consistently associated with lower postoperative lactate and creatinine, fewer bleeding complications, and better survival trajectories.

The observation that DL support reduces lactate and creatinine suggests protection of 2 vital systems: tissue perfusion and renal function. These biomarkers are dynamic indicators of systemic support in the acute phase, not just markers of severity. This aligns with growing recognition that lactate is a key signal of patient trajectory during ECLS, able to guide support strategies and identify those who respond to treatment.^21^ Early improvement in perfusion and renal function may increase the chance of reversing shock and preventing irreversible organ injury.^22^^,^^23^

The key clinical insight of this study is that DL cannulation does not act by directly reducing mortality but by modifying the physiological conditions that drive outcomes. DL provides more effective venous drainage and reduces recirculation, thereby improving oxygen delivery and stabilizing ventricular loading conditions.^24^^,^^25^

Rather than being only a technical option, DL cannulation serves as a protective strategy in the vulnerable early phase of RVF. By improving perfusion and renal function, it creates a more favorable setting for recovery. The absence of a direct mortality effect in the statistical models, combined with strong indirect pathways through organ function, supports this interpretation. Importantly, the benefit of DL was consistent across different cannulation sites and was not affected by the use of an oxygenator, whereas patients with lower baseline Pao_2_ derived the greatest advantage.

Phenotypic clustering provided an additional clinical perspective. Postoperative biochemical patterns, derived from routine markers, identified subgroups of patients with very different prognoses. Patients with low lactate and preserved renal function had excellent outcomes, whereas those with tissue hypoperfusion and renal dysfunction fared poorly. Importantly, DL cannulation shifted patients toward the favorable phenotypes.^26^

This suggests that patient profile after ECLS is not fixed but can be modified by the choice of cannulation strategy. In this light, DL should not be seen only as a technical alternative but as an intervention capable of influencing early biological responses and, ultimately, clinical outcomes.

These results support a more physiology-driven approach to cannulation choice. If early physiology determines outcomes, and if DL cannulation can improve perfusion and renal function, then the decision should aim to maximize early organ protection. Patients with hypoxemia, venous congestion, or impaired renal reserve appear to benefit the most from DL support.^27^^,^^28^

Success should therefore not be judged only by survival but also by whether the chosen strategy optimized lactate clearance, preserved renal function, and shifted patients away from high-risk profiles. This represents a step toward personalized ECMO, where device configuration is selected according to patient physiology rather than operator preference.^29^

Earlier studies mainly demonstrated feasibility and safety, but rarely addressed the mechanisms or the patients most likely to benefit. Our work is the first to combine mediation analysis and clustering to clarify both how and in whom DL cannulation confers an advantage. This distinguishes it from previous large observational cohorts that considered cannulation as a simple technical choice, without exploring underlying physiology or patient subgroups.^30^

Our findings could reposition DL cannulation not merely as a venous access option but as a physiological tool capable of improving organ function, reducing complications, and enhancing recovery. This perspective might open the way for a more personalized use of ECMO in RVF, guided by clinical and physiological markers.

### Limitations

This study has limitations. Although based on prospectively collected registry data with robust adjustment, the observational design cannot eliminate residual confounding. Mediation and clustering relied on early postoperative biomarkers and may not capture late events. Classification into biochemical phenotypes, although reproducible, requires external validation in broader ECLS populations.

Cannulation site did not show effect modification, yet confounding by procedural selection cannot be excluded. Oxygenator use could not be standardized across centers; although nonsignificant in interaction models, it remains a marker of severity and residual confounding is possible. Finally, institutional, and operator-dependent factors such as weaning protocols and timing may have influenced outcomes.

### Declaration of Generative AI and AI-Assisted Technologies in the Writing Process

During the preparation of this work the authors used OpenAI ChatGPT for language editing and DALL·E for original figures. After using this tool/service, the authors reviewed and edited the content as needed and take(s) full responsibility for the content of the publication.

## Conflict of Interest Statement

D. Wiedemann reported consultant/proctor for Abbott and scientific advisor for Xenios/Fresenius. T. Verbelen reported consultant for Medtronic. L.M. Broman reported medical advisory board for Eurosets Srl and for Xenios AG. F. Pappalardo reported consultant for Abiomed. R. Lorusso reported consultant for Medtronic, LivaNova, Getinge, and Abiomed and serves as medical advisory board member for Eurosets and Xenios. All other authors reported no conflicts of interest.

The *Journal* policy requires editors and reviewers to disclose conflicts of interest and to decline handling or reviewing manuscripts for which they may have a conflict of interest. The editors and reviewers of this article have no conflicts of interest.

## Supplementary Data

Figure E1

Figure E2

Figure E3

Figure E4

Figure E5

Figure E6

Figure E7

Figure E8

Figure E9

Figure E10

Figure E11

Figure E12

Figure E13

Figure E14

Figure E15
