# Corrigendum to ‘Evaluation of dual-lumen pulmonary artery cannulation in extracorporeal right ventricular support’ [JTCVS Open. 2026;30:101699]

**DOI:** 10.1016/j.xjon.2026.102058

**Published:** 2026-08-13

**Authors:** Valeria Lo Coco, Michele Di Mauro, Silvia Mariani, Elham Bidar, Kasia Hryniewicz, Antonio Loforte, Thomas Fux, Sam Heuts, Dominik Wiedemann, Michal Kawcynsky, Tom Verbelen, Lars Mikael Broman, Jamila Kremer, Matteo Pozzi, Koji Takeda, Udo Boeken, Yih-Sharng Chen, Paolo Masiello, Dominik J. Vogel, Jacinta J. Maas, Andrea Ballotta, Federico Pappalardo, Sandro Gelsomino, Roberto Lorusso

**Affiliations:** a Cardio-Thoracic Surgery Department, Maastricht University Medical Center, Maastricht, The Netherlands; b Cardiology Department, Minneapolis Heart Institute, Minneapolis, Minn; c Cardiac Surgery Department, Sant’Orsola University Hospital, Bologna, Italy; d Molecular Medicine and Surgery Department, Karolinska Institute, Stockholm, Sweden; e Perioperative Medicine and Intensive Care Department, Karolinska University Hospital, Stockholm, Sweden; f Cardiac Surgery Department, Medical University of Vienna, Vienna, Austria; g Cardiac Surgery Department, University Hospital Leuven, Leuven, Belgium; h Intensive Care Unit, ECMO Center Karolinska, Karolinska University Hospital, Stockholm, Sweden; i Department of Physiology and Pharmacology, Karolinska Institutet, Stockholm, Sweden; j Cardiac Surgery Department, University Hospital, Heidelberg, Germany; k Cardiac Surgery Department, Louis Pradel Cardiologic Hospital, Lyon, France; l Cardiac Surgery Department, Columbia University Irving Medical Center, New York, NY; m Cardiac Surgery Department, University Hospital, Düsseldorf, Germany; n Cardiovascular Surgery Department, National University Hospital, Taipei City, Taiwan; o Emergency Cardiac Surgery - Cardio-Thoracic-Vascular Department, University Hospital San Giovanni di Dio e Ruggi D'Aragona, Salerno, Italy; p Intensive Care Medicine Department, Guy's and St Thomas' NHS Foundation Trust, London, United Kingdom; q Intensive Care Department, Leiden University Medical Center, Leiden, The Netherlands; r Intensive Care Unit Department, IRCCS Centro Cardiologico Monzino, Milano, Italy; s Cardiothoracic and Vascular Anesthesia and Intensive Care Department, SS Antonio e Biagio e Cesare Arrigo Hospital, Alessandria, Italy; t Cardiovascular Research Institute Maastricht (CARIM), Maastricht, The Netherlands

The authors regret that in the version of this article originally published, the reference list did not correspond to the sources actually consulted and intended by the authors. A generative artificial intelligence tool was used during the preparation of the manuscript, and its use was declared at submission. A number of the citations returned by that tool were inaccurate or did not correspond to existing publications, and this was not identified by the authors during internal revision or at the proof stage.

Following publication, the authors re-checked every citation individually against the primary sources and against the corresponding PubMed and Crossref records, and rebuilt the reference list accordingly. The affected items are the reference list and the numbering of the in-text citations. The study population, the data, the statistical analyses, the results, the figures, the tables and the conclusions of the article are unchanged and are not affected by this error.

The corrected reference list is given below and replaces in full the reference list of the published version; the in-text citation numbers are those indicated above. The authors apologise to the readers, to the Editors and to the Journal for this error, for which the corresponding author takes full responsibility.

The authors would like to apologise for any inconvenience caused.
