# Supplementary figures and images for: Evaluation of dual-lumen pulmonary artery cannulation in extracorporeal right ventricular support
Source: JTCVS Open. 2026 Mar 4;30:101699. doi: 10.1016/j.xjon.2026.101699 (PMC13131193; doi:10.1016/j.xjon.2026.101699)

In-Hospital Mortality by Cannula type

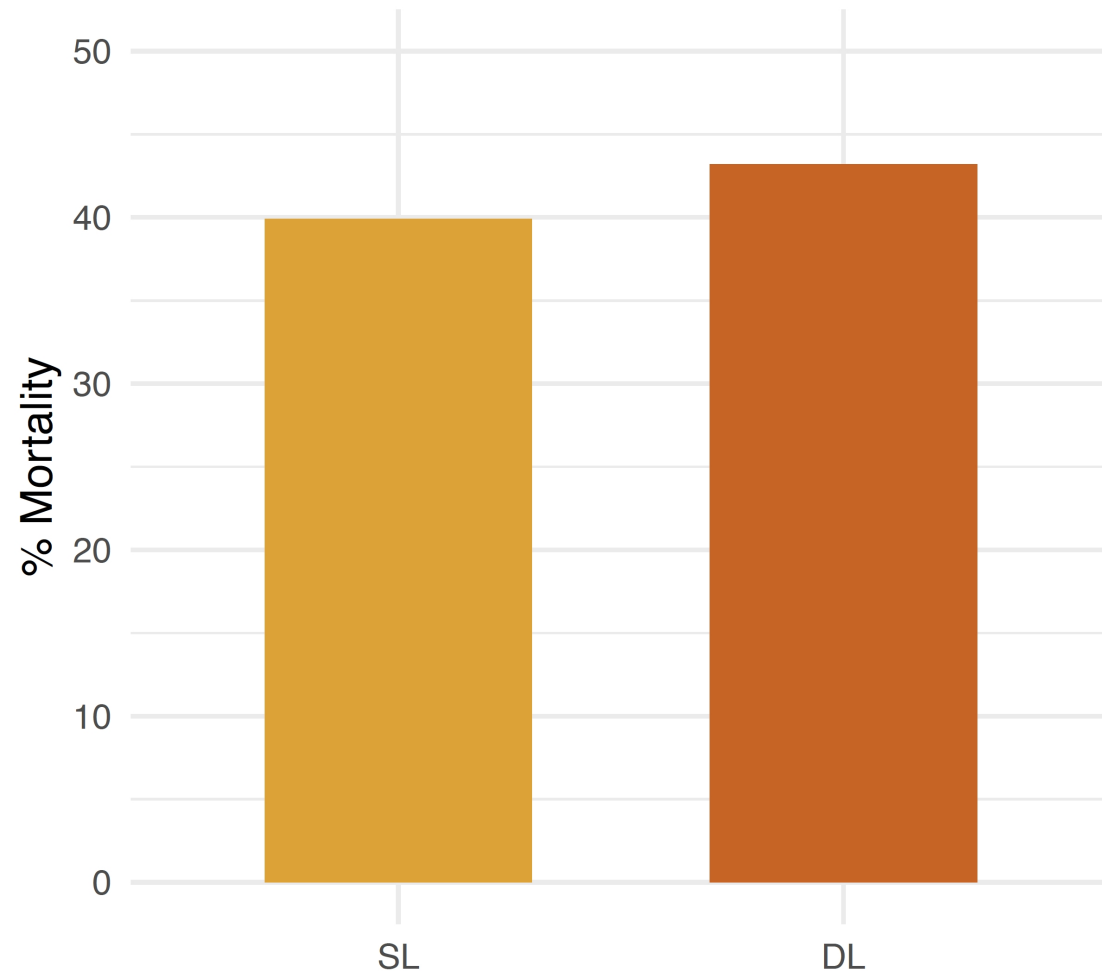

30-day Mortality by Cannula type

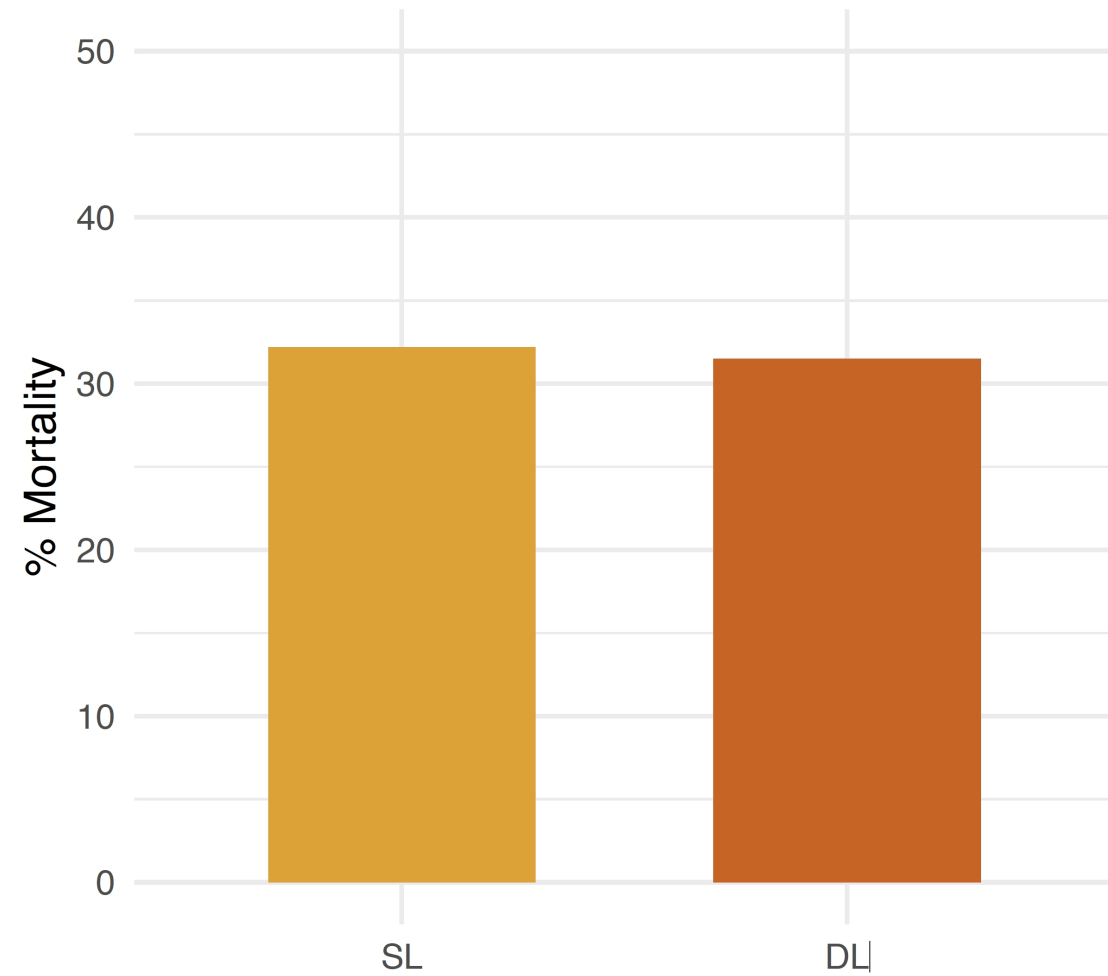

Supplement: Figure E1 [file mmc1.pdf]

Pre-weighting

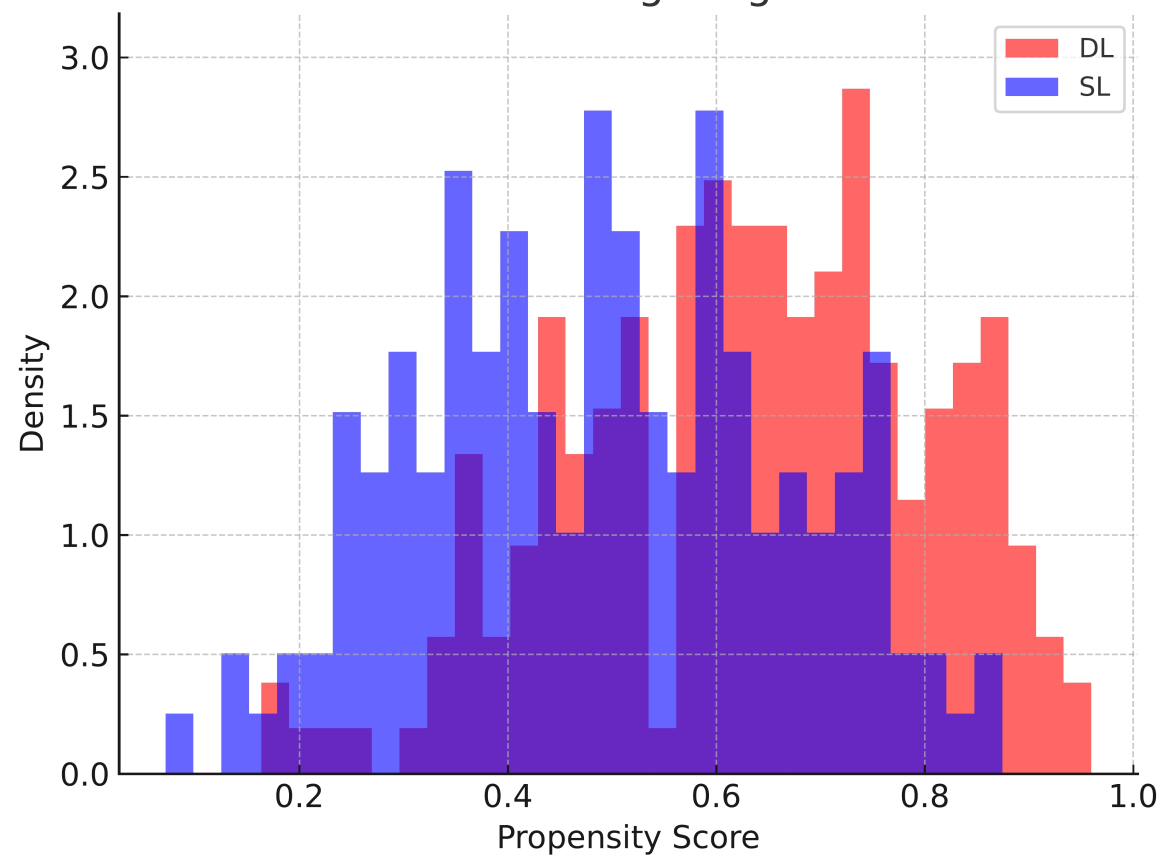

Post-weighting

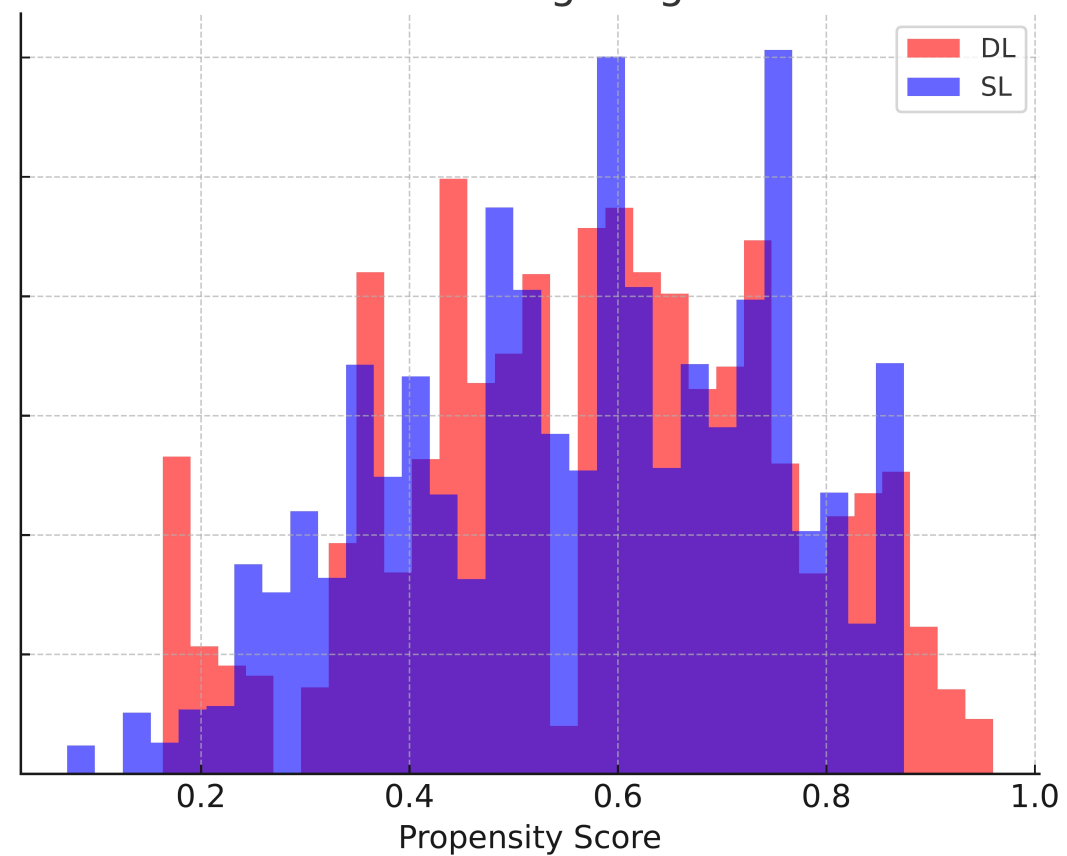

Supplement: Figure E5 [file mmc5.pdf]

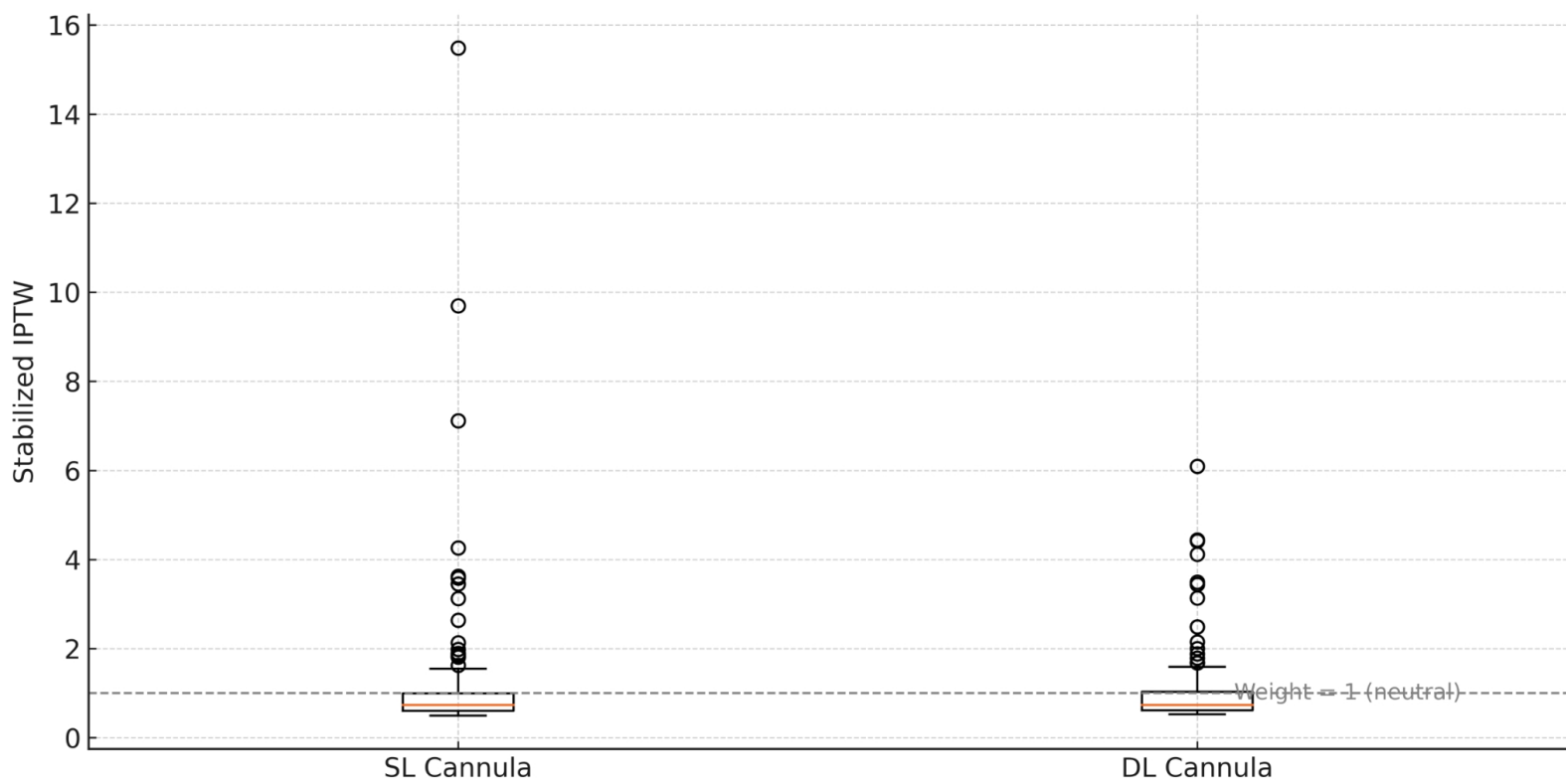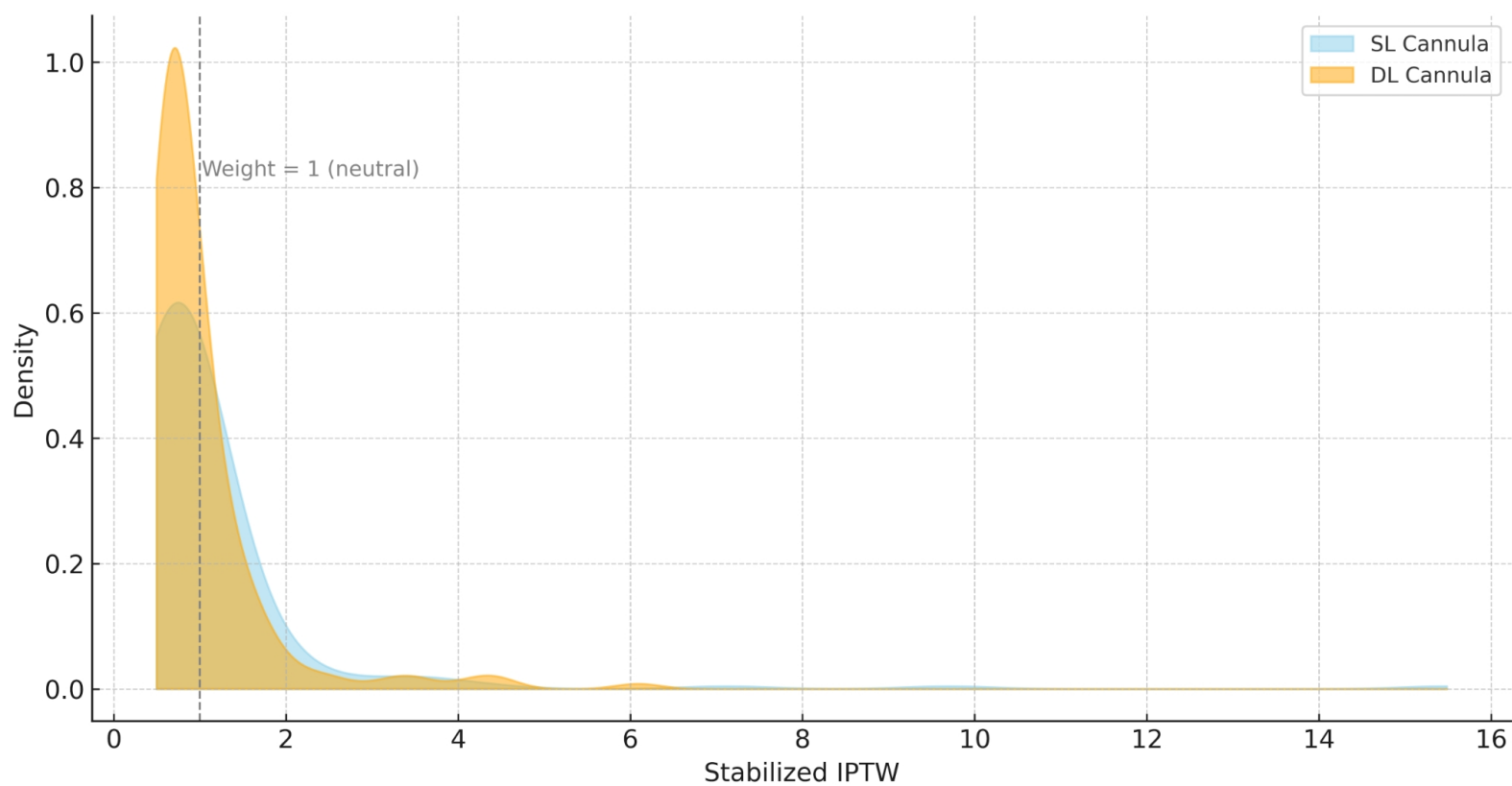

Supplement: Figure E6 [file mmc6.pdf]

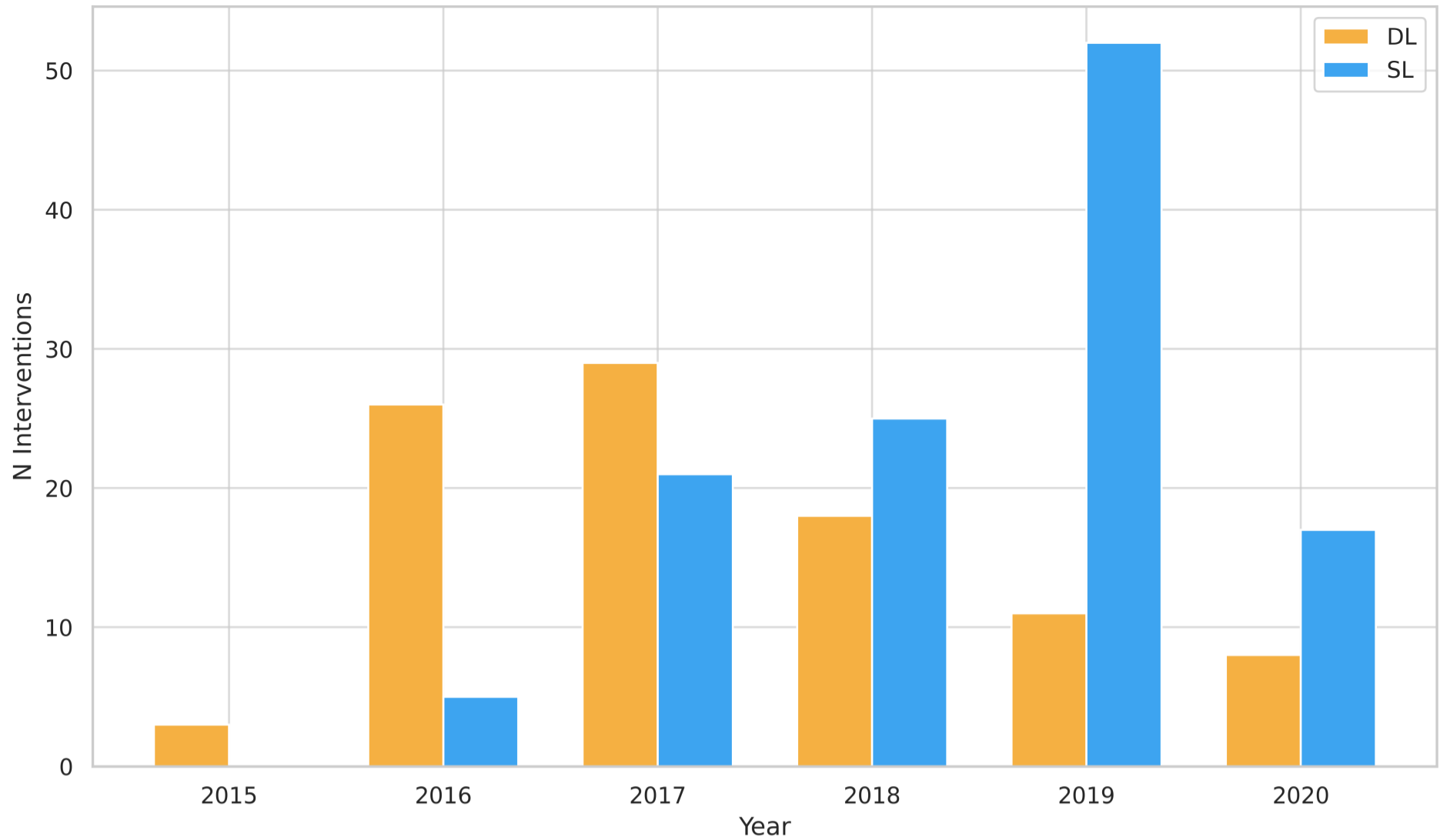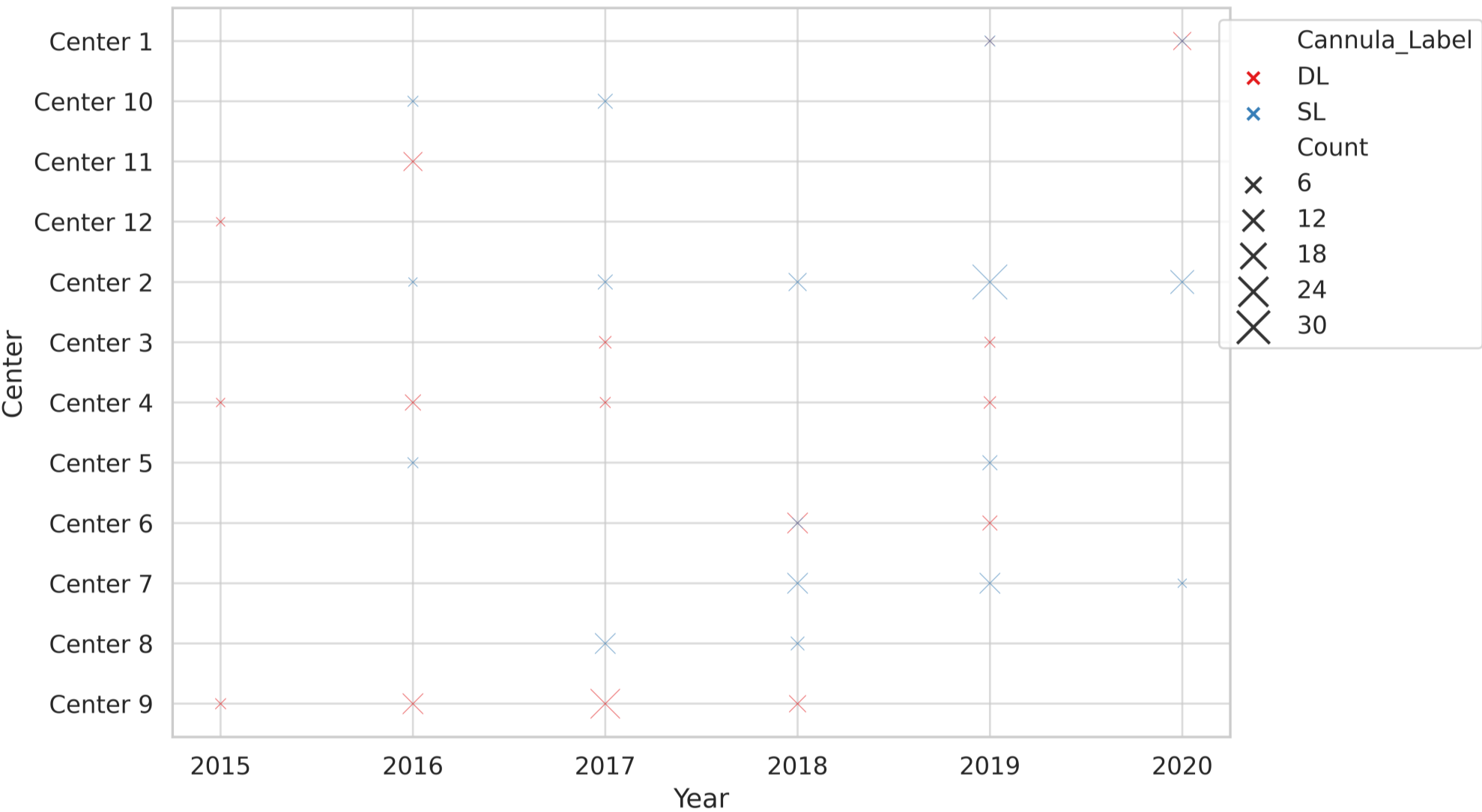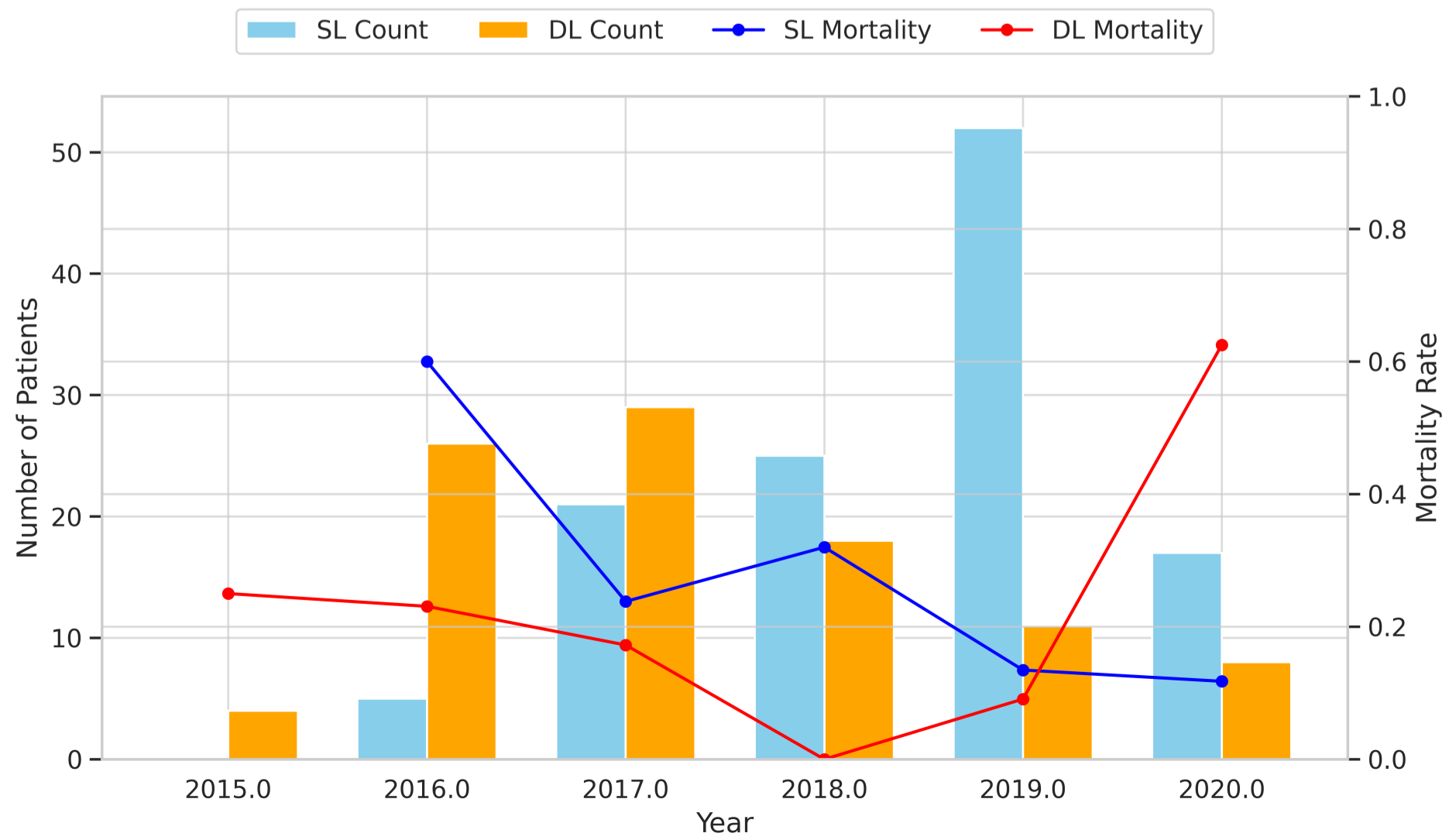

Supplement: Figure E8 [file mmc8.pdf]

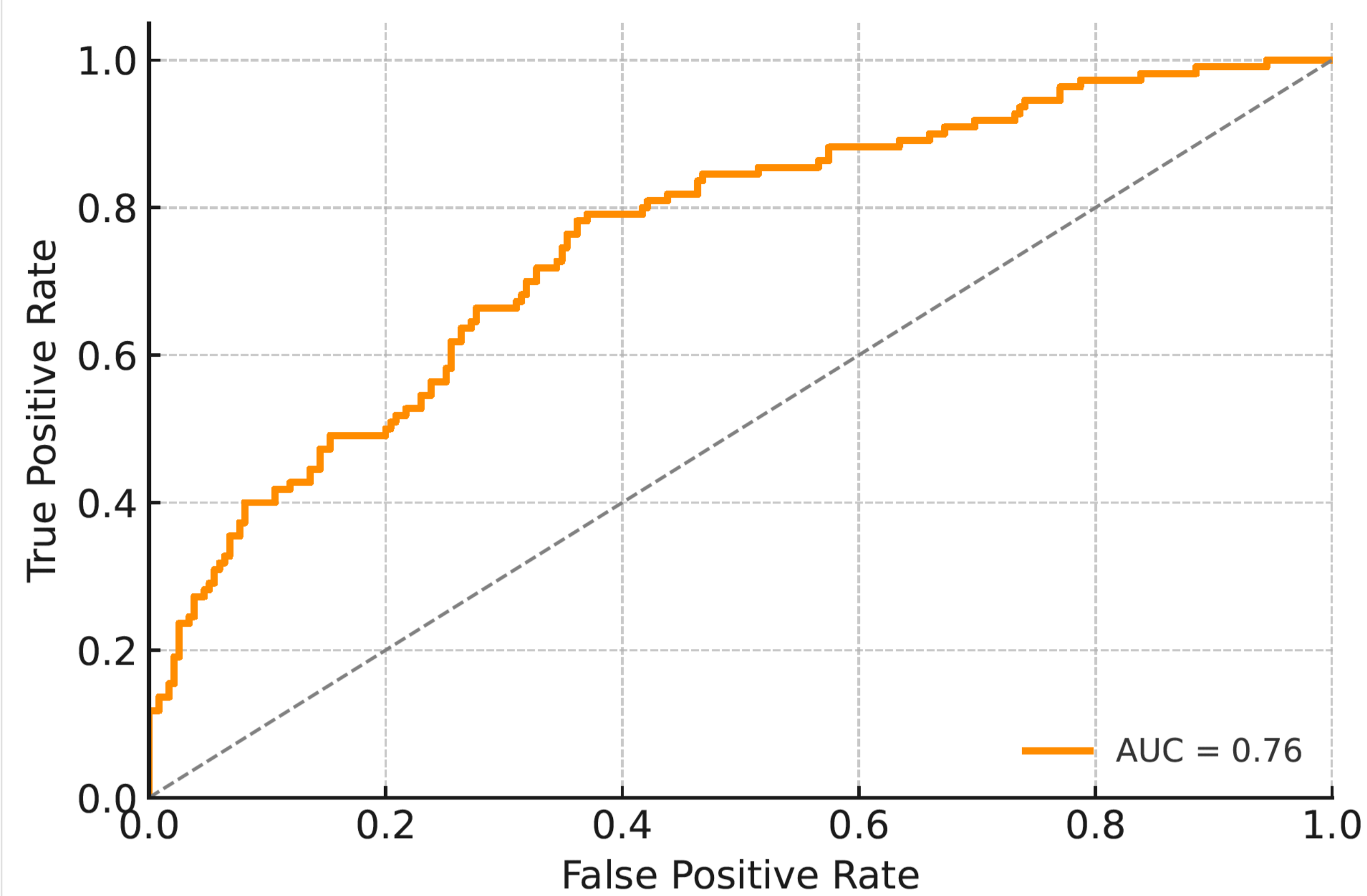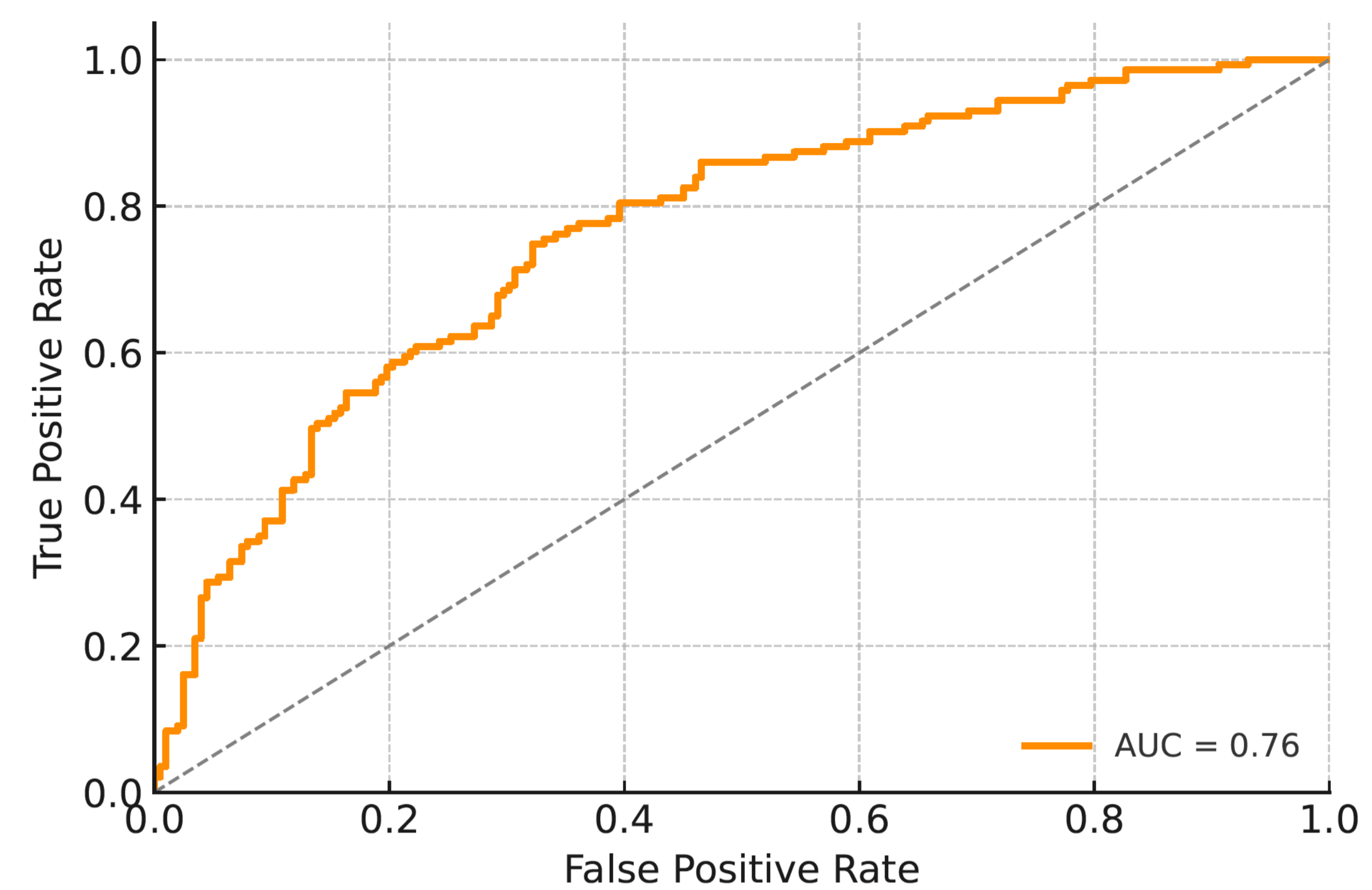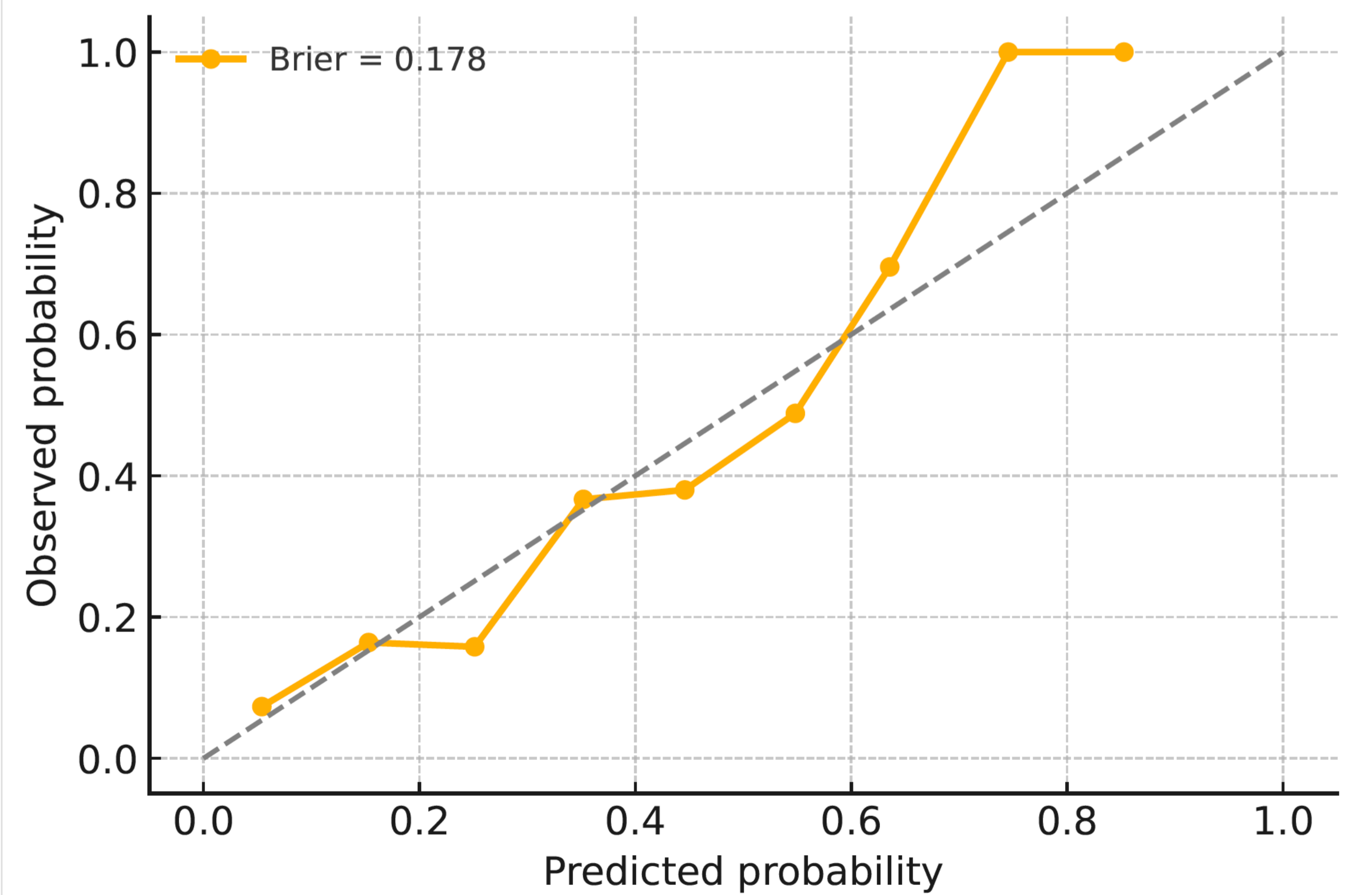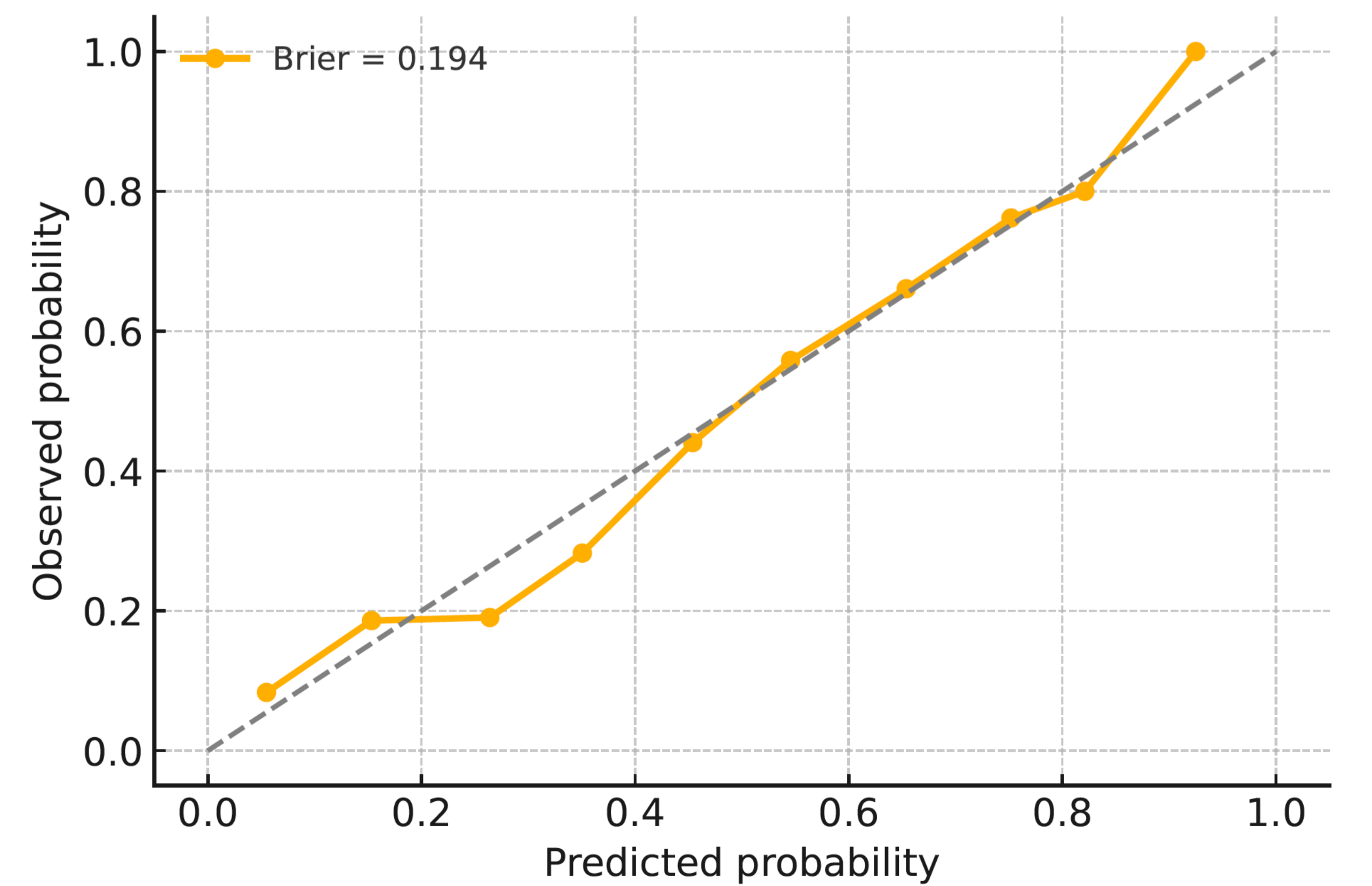

Supplement: Figure E9 [file mmc9.pdf]

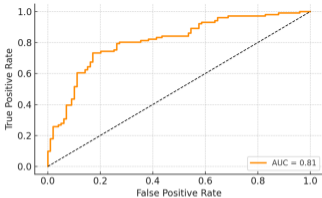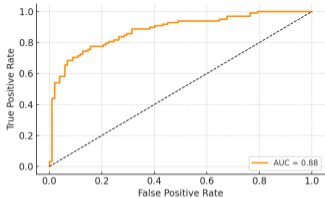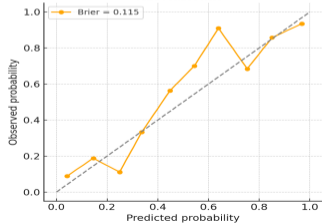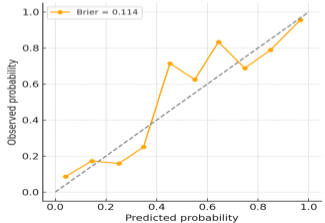

Supplement: Figure E10 [file mmc10.pdf]

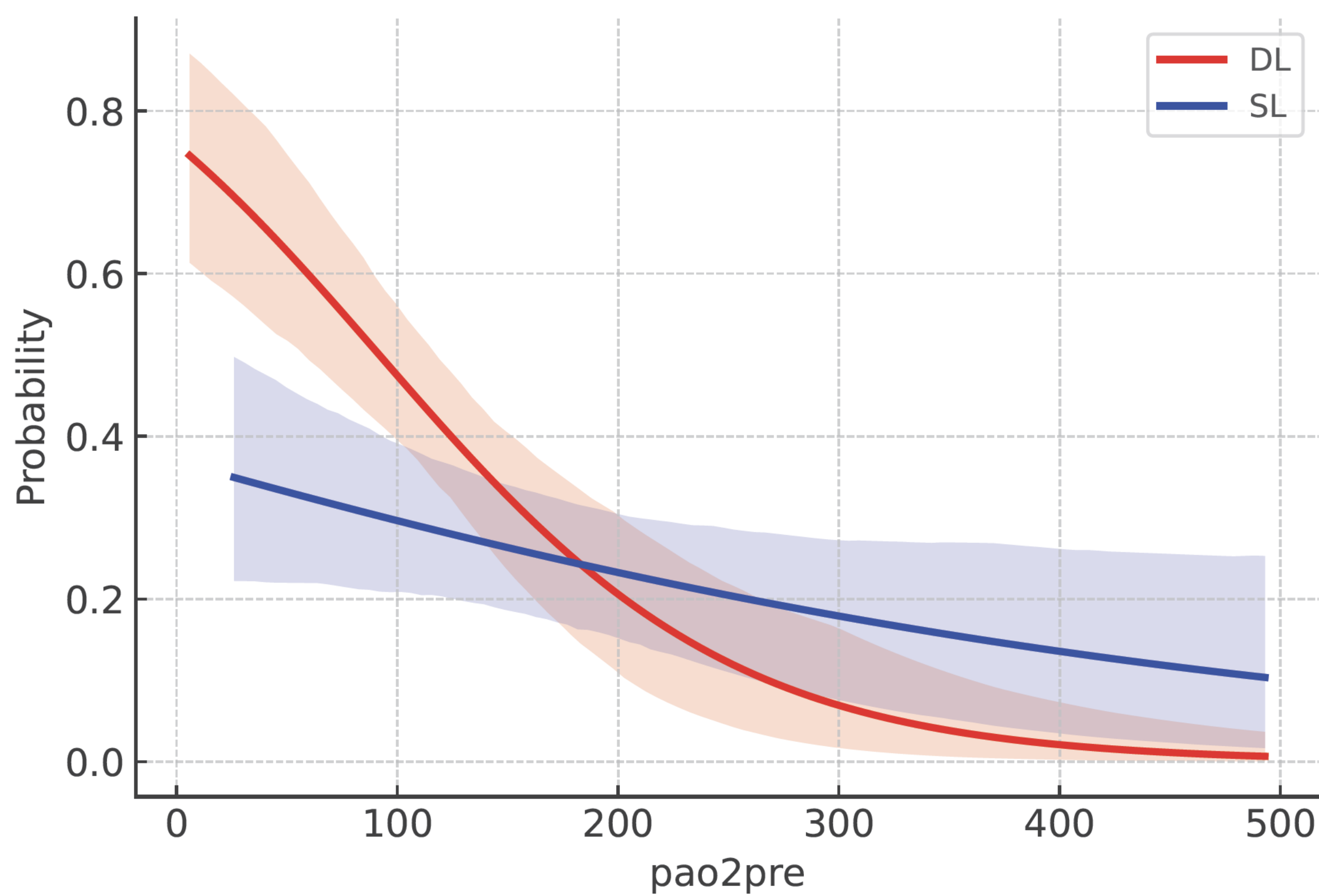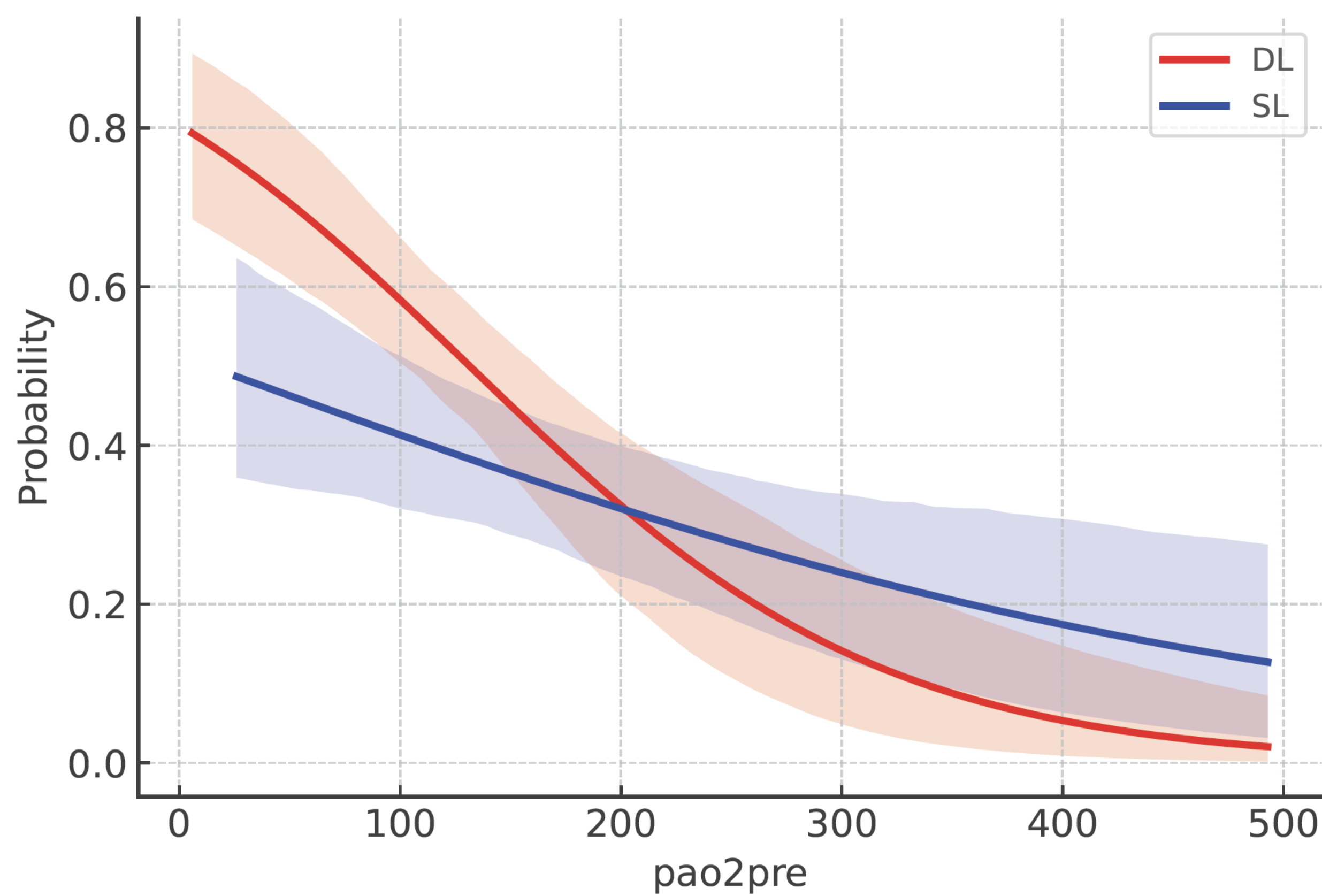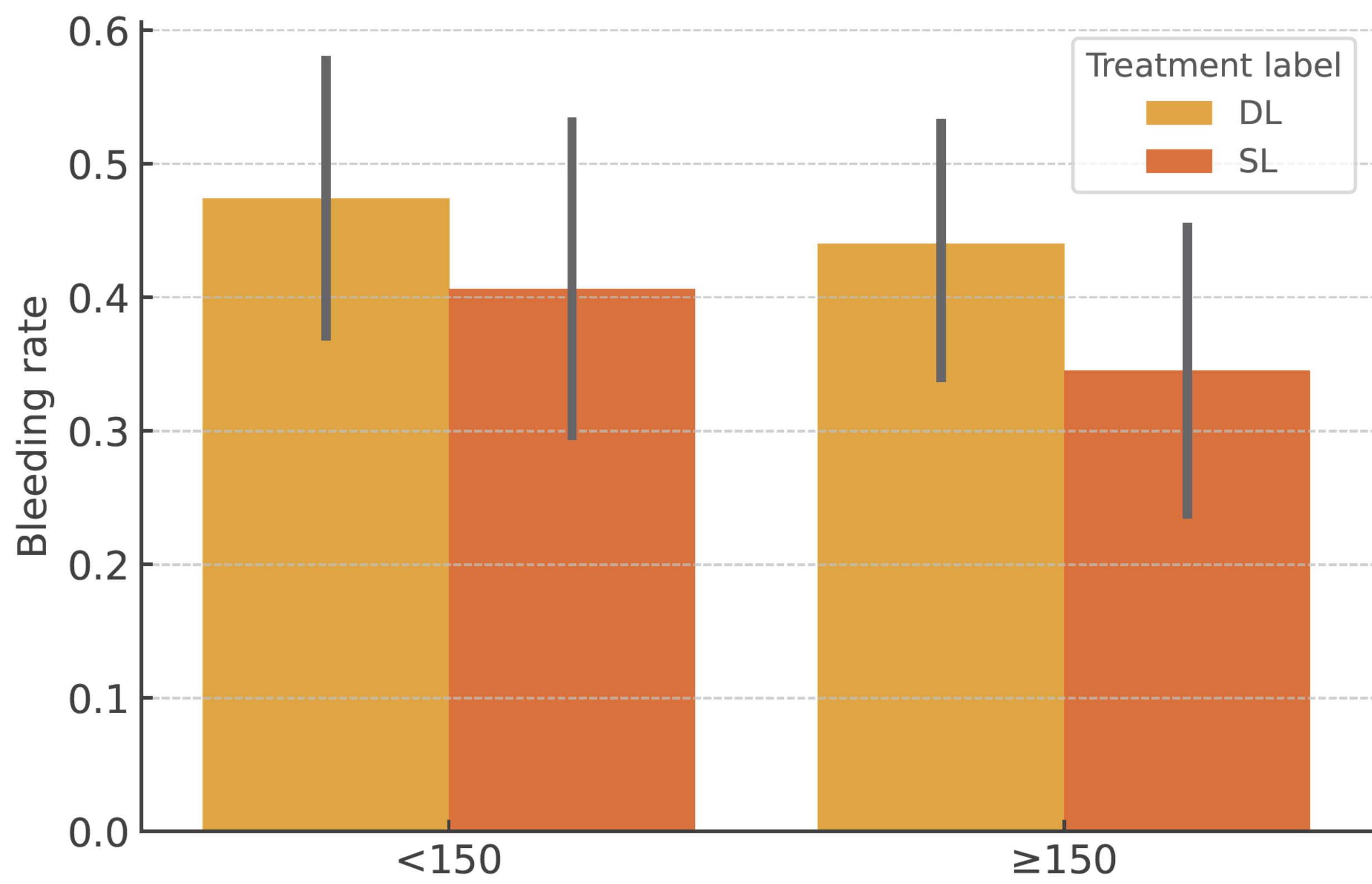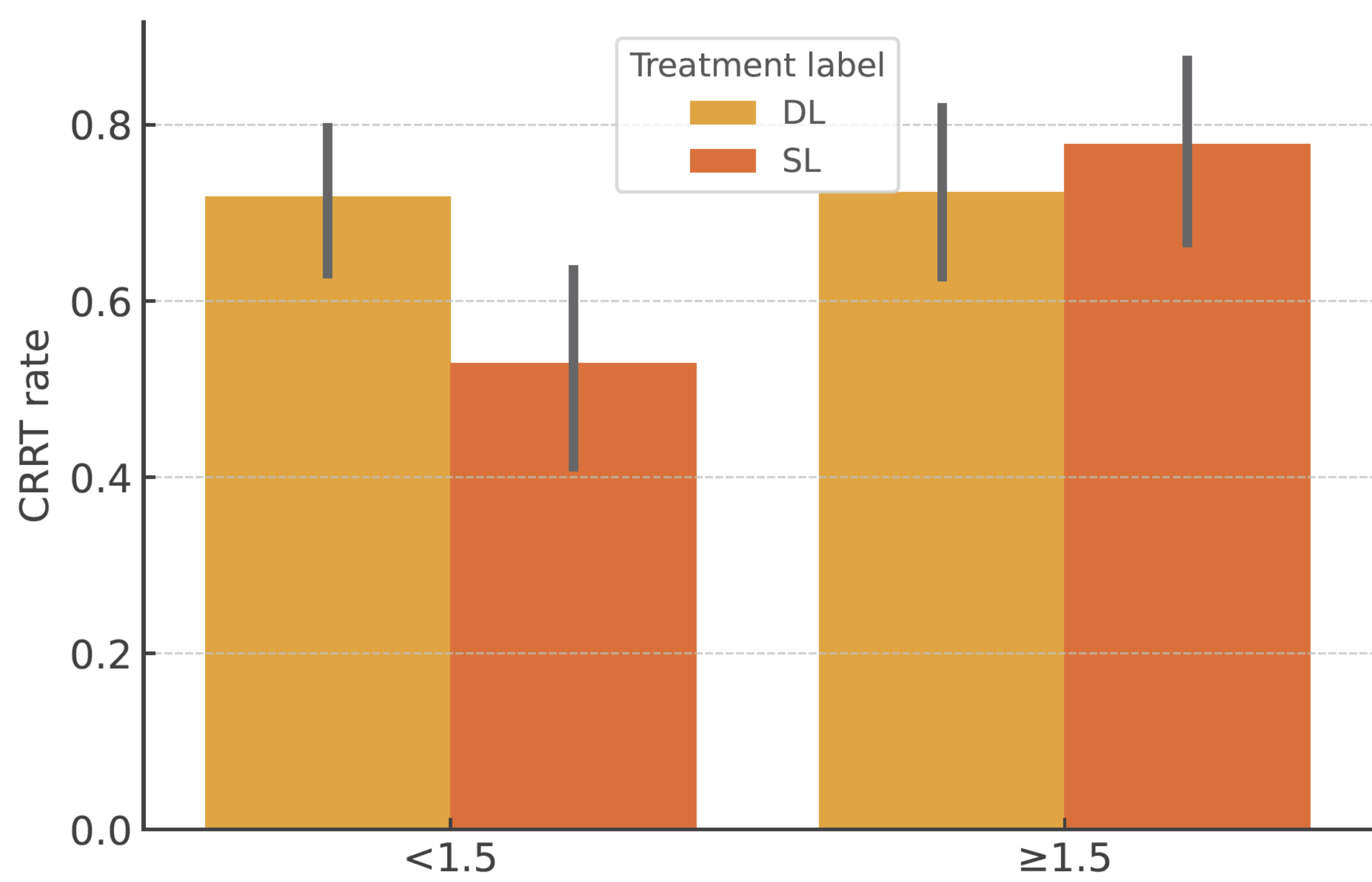

Supplement: Figure E11 [file mmc11.pdf]

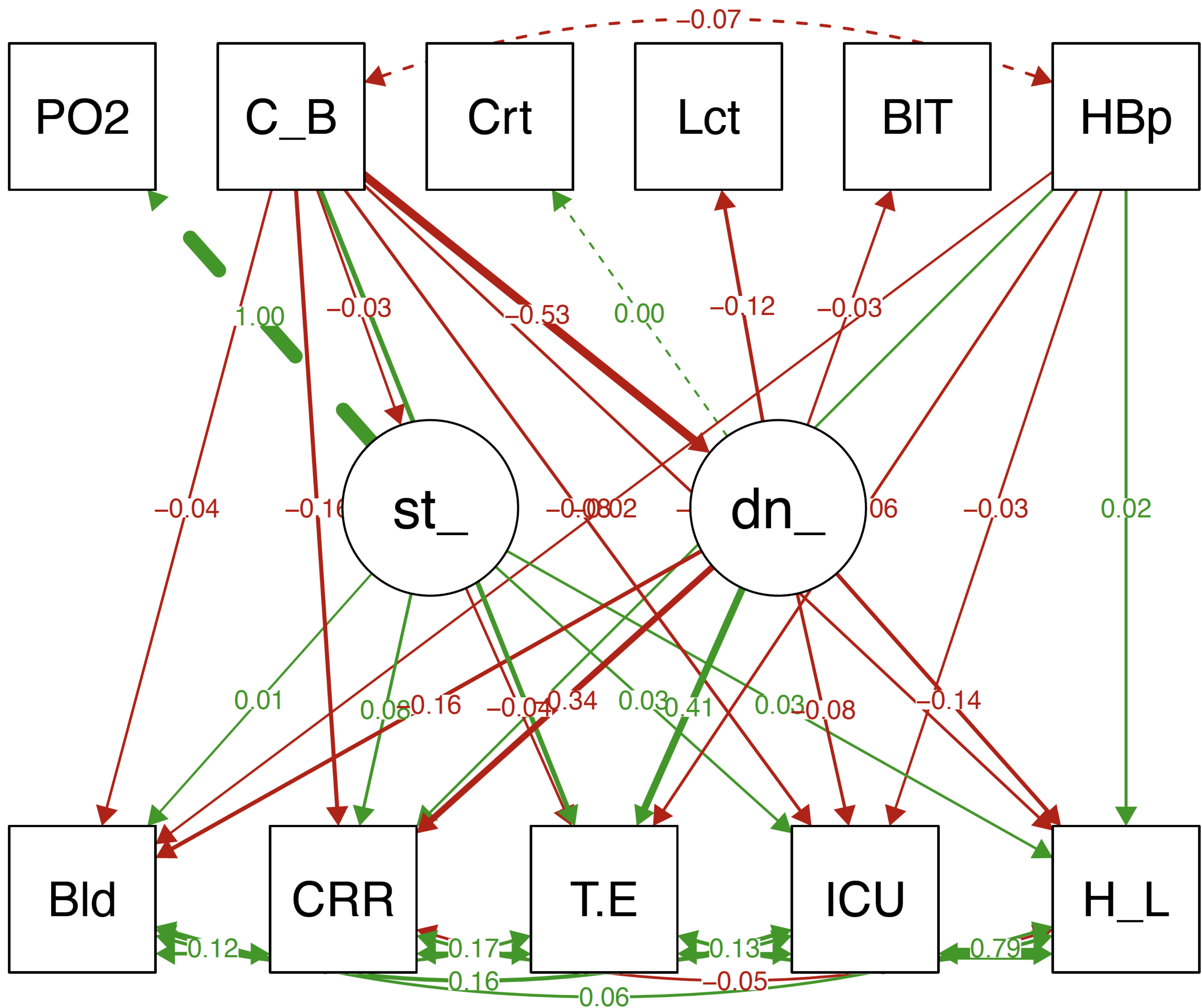

Supplement: Figure E12 [file mmc12.pdf]

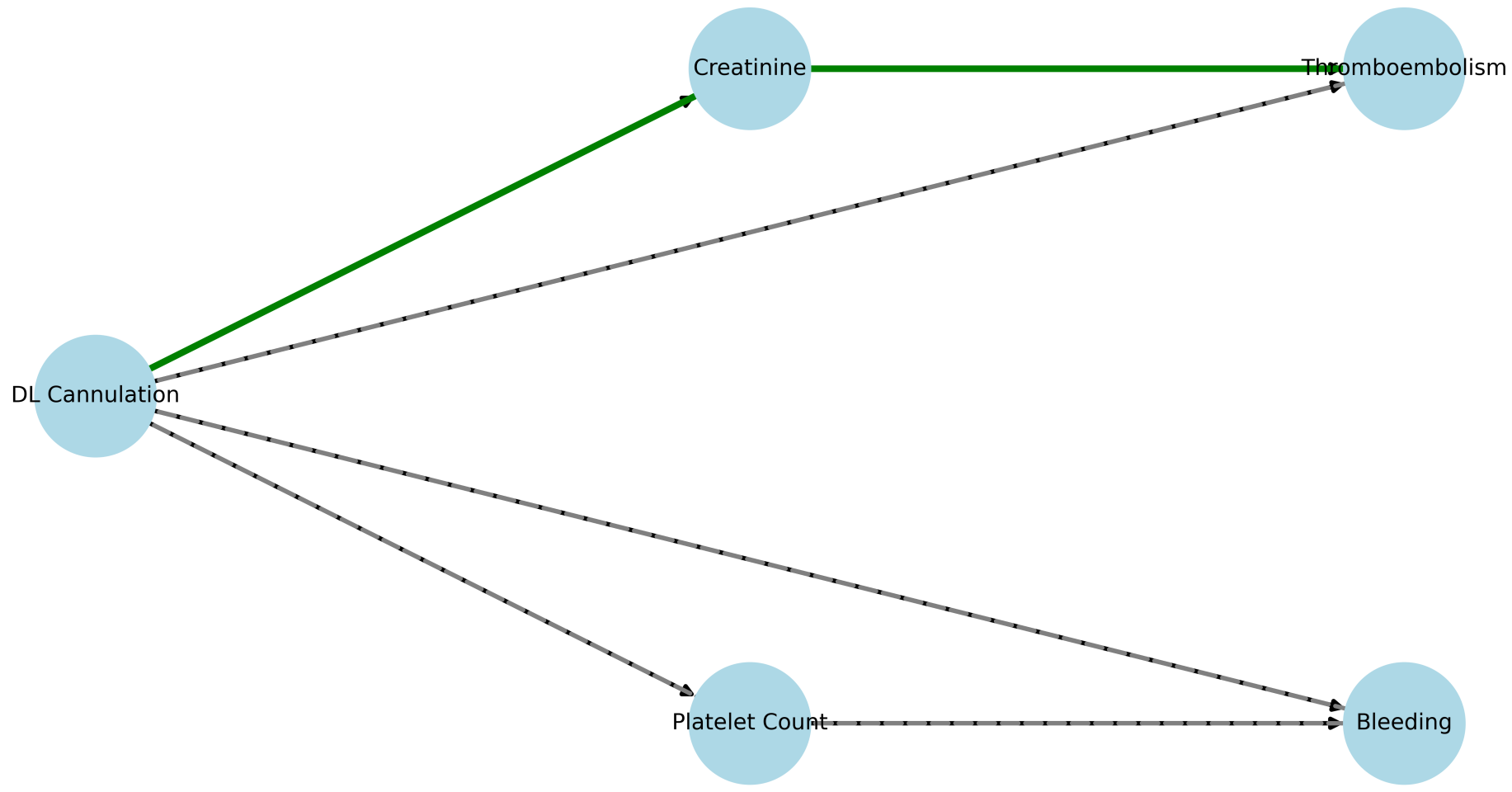

Supplement: Figure E13 [file mmc13.pdf]

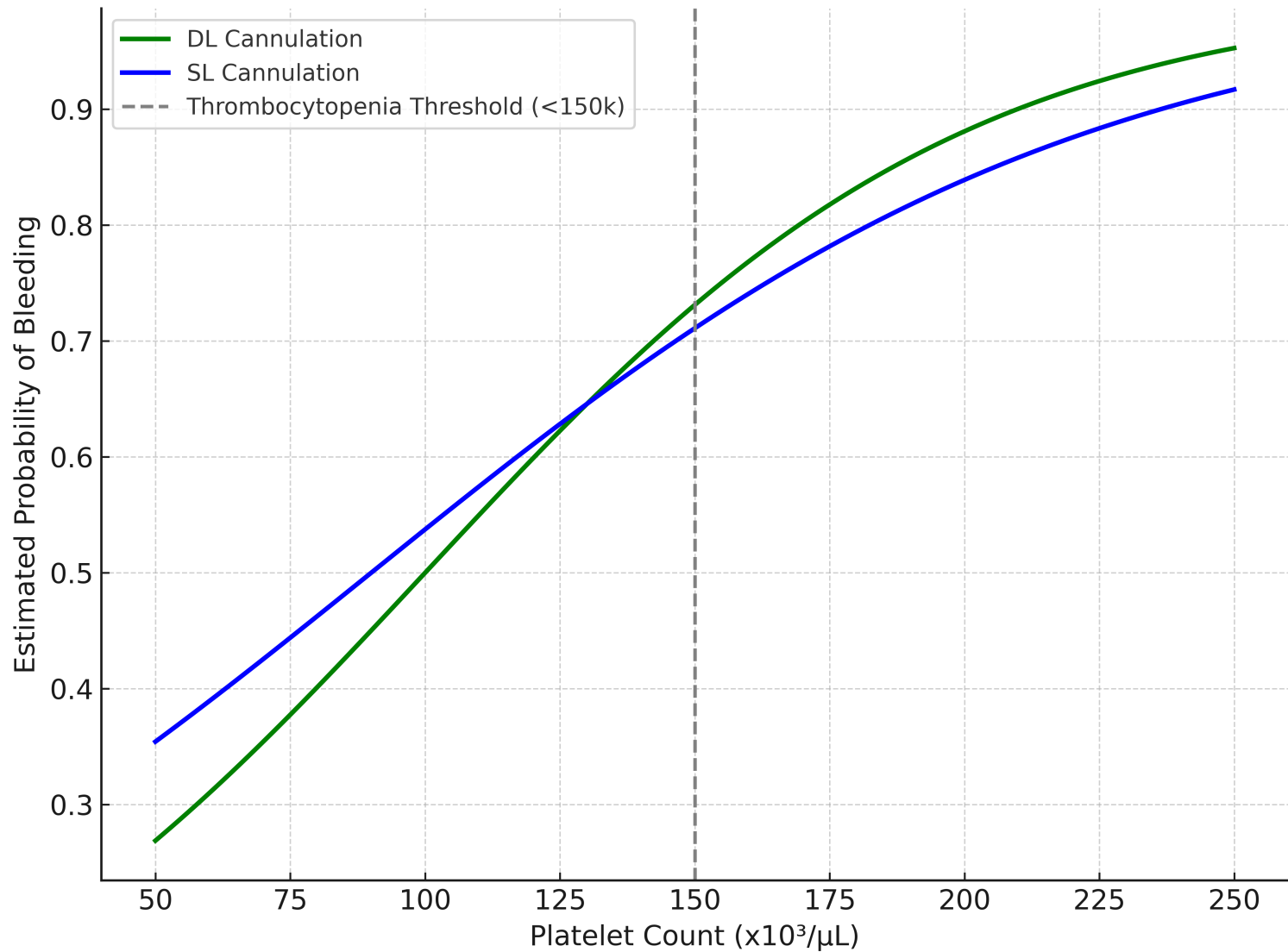

Supplement: Figure E14 [file mmc14.pdf]
