# Supplementary material for: Evaluation of dual-lumen pulmonary artery cannulation in extracorporeal right ventricular support
Source: JTCVS Open. 2026 Mar 4;30:101699. doi: 10.1016/j.xjon.2026.101699 (PMC13131193; doi:10.1016/j.xjon.2026.101699)
Supplement: Figure E2 [file mmc2.pdf]

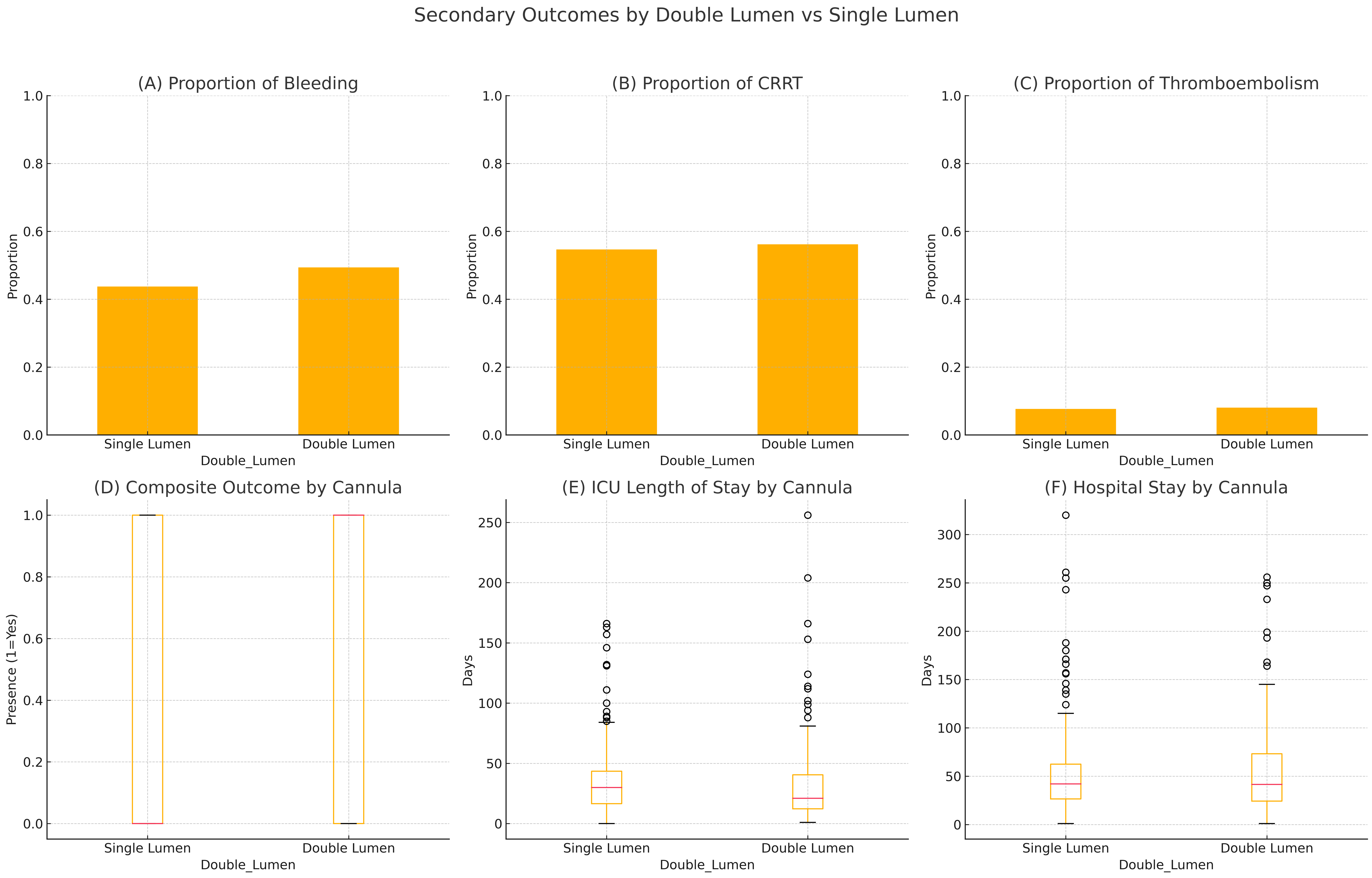

Secondary Outcomes by Double Lumen vs Single Lumen
(A) Proportion of Bleeding
(B) Proportion of CRRT
(C) Proportion of Thromboembolism
(D) Composite Outcome by Cannula
(E) ICU Length of Stay by Cannula
(F) Hospital Stay by Cannula
Proportion
Presence (1=Yes)
Days
Single Lumen
Double Lumen
Double_Lumen
0.0
0.2
0.4
0.6
0.8
1.0
0
50
100
150
200
250
300
