# Supplementary material for: Evaluation of dual-lumen pulmonary artery cannulation in extracorporeal right ventricular support
Source: JTCVS Open. 2026 Mar 4;30:101699. doi: 10.1016/j.xjon.2026.101699 (PMC13131193; doi:10.1016/j.xjon.2026.101699)
Supplement: Figure E3 [file mmc3.pdf]

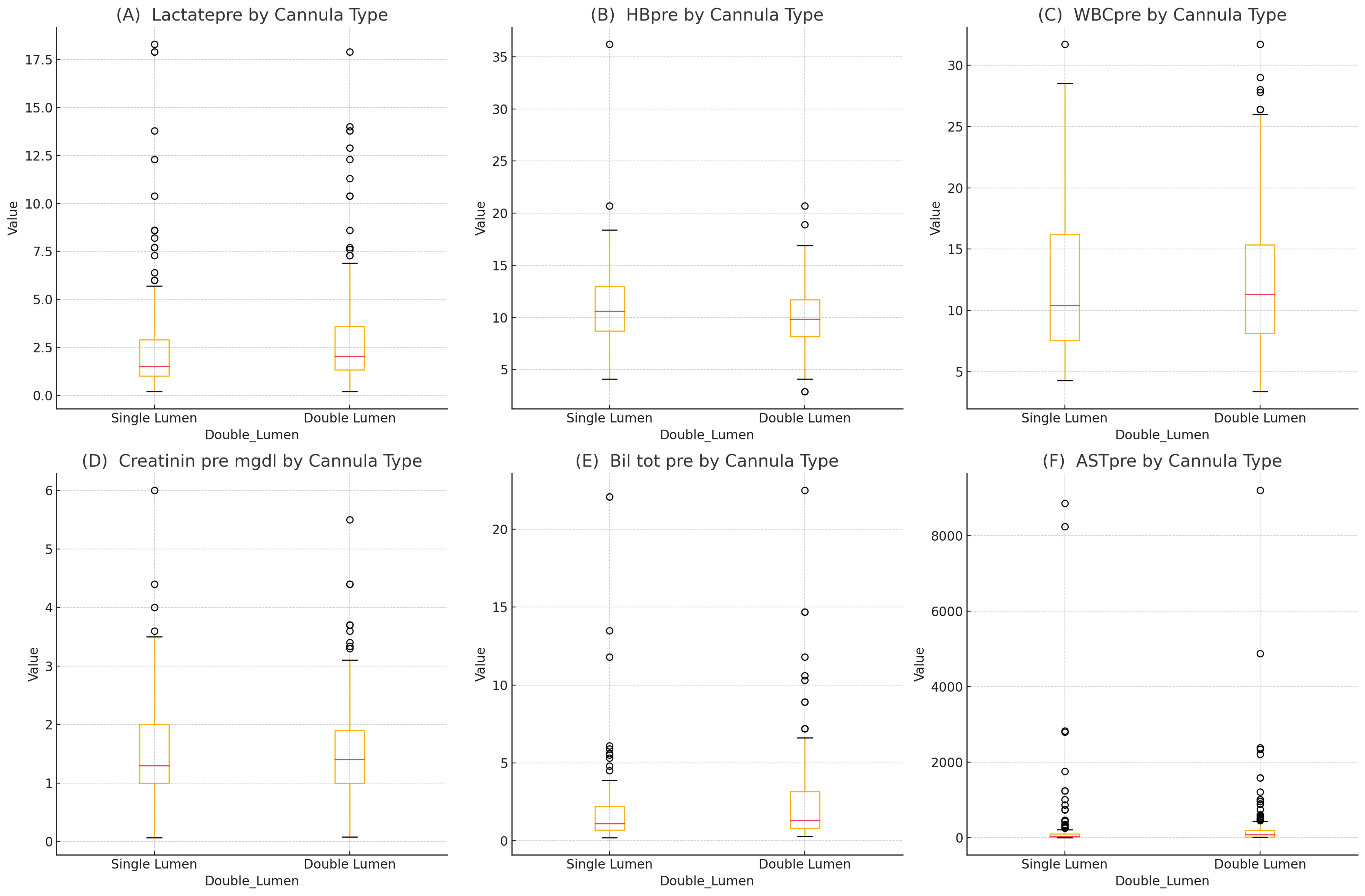

(A) Lactatepre by Cannula Type
(B) HBpre by Cannula Type
(C) WBCpre by Cannula Type
(D) Creatinin pre mgdl by Cannula Type
(E) Bil tot pre by Cannula Type
(F) ASTpre by Cannula Type
Value
Single Lumen
Double Lumen
Double_Lumen
0.0
2.5
5.0
7.5
10.0
12.5
15.0
17.5
5
10
15
20
25
30
35
0
1
2
3
4
5
6
0
2000
4000
6000
8000
