# Supplementary material for: Evaluation of dual-lumen pulmonary artery cannulation in extracorporeal right ventricular support
Source: JTCVS Open. 2026 Mar 4;30:101699. doi: 10.1016/j.xjon.2026.101699 (PMC13131193; doi:10.1016/j.xjon.2026.101699)
Supplement: Figure E4 [file mmc4.pdf]

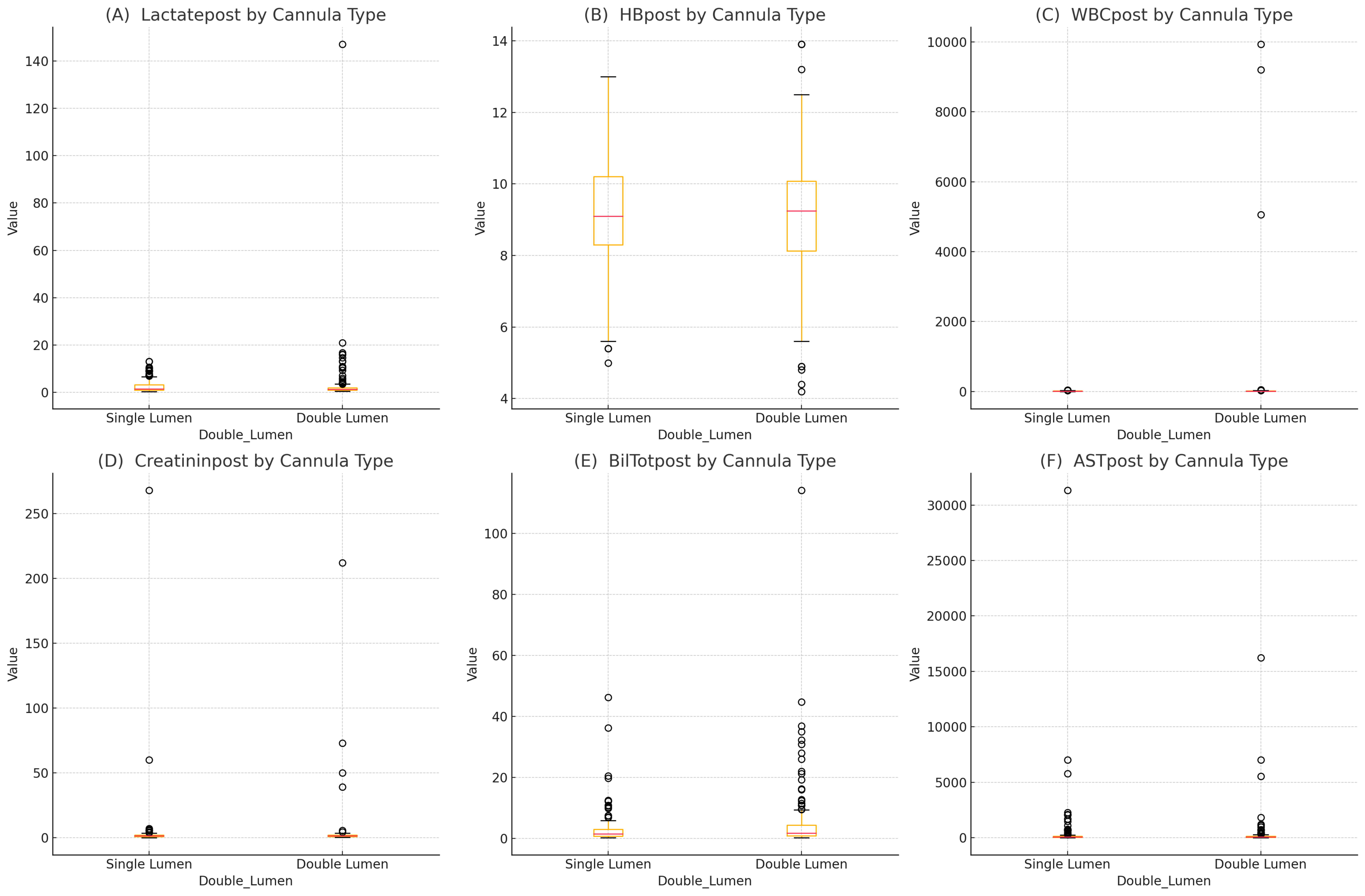

(A) Lactatepost by Cannula Type
(B) HBpost by Cannula Type
(C) WBCpost by Cannula Type
(D) Creatininpost by Cannula Type
(E) BilTotpost by Cannula Type
(F) ASTpost by Cannula Type
Value
Single Lumen
Double Lumen
Double_Lumen
