# Supplementary material for: Evaluation of dual-lumen pulmonary artery cannulation in extracorporeal right ventricular support
Source: JTCVS Open. 2026 Mar 4;30:101699. doi: 10.1016/j.xjon.2026.101699 (PMC13131193; doi:10.1016/j.xjon.2026.101699)
Supplement: Figure E7 [file mmc7.pdf]

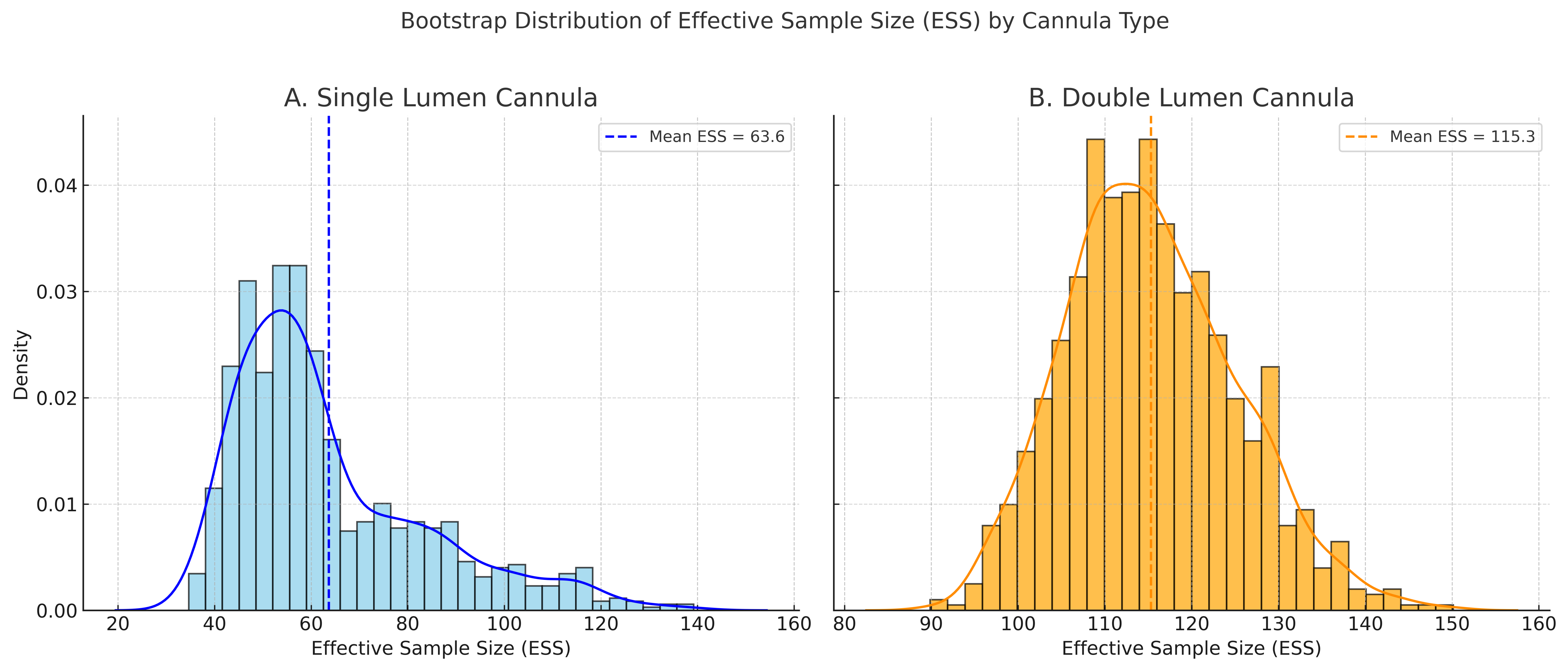

Bootstrap Distribution of Effective Sample Size (ESS) by Cannula Type
A. Single Lumen Cannula
Mean ESS = 63.6
B. Double Lumen Cannula
Mean ESS = 115.3
Density
0.00
0.01
0.02
0.03
0.04
20
40
60
80
100
120
140
160
90
110
130
150
Effective Sample Size (ESS)
Effective Sample Size (ESS)
