# Supplementary material for: Evaluation of dual-lumen pulmonary artery cannulation in extracorporeal right ventricular support
Source: JTCVS Open. 2026 Mar 4;30:101699. doi: 10.1016/j.xjon.2026.101699 (PMC13131193; doi:10.1016/j.xjon.2026.101699)
Supplement: Figure E15 [file mmc15.pdf]

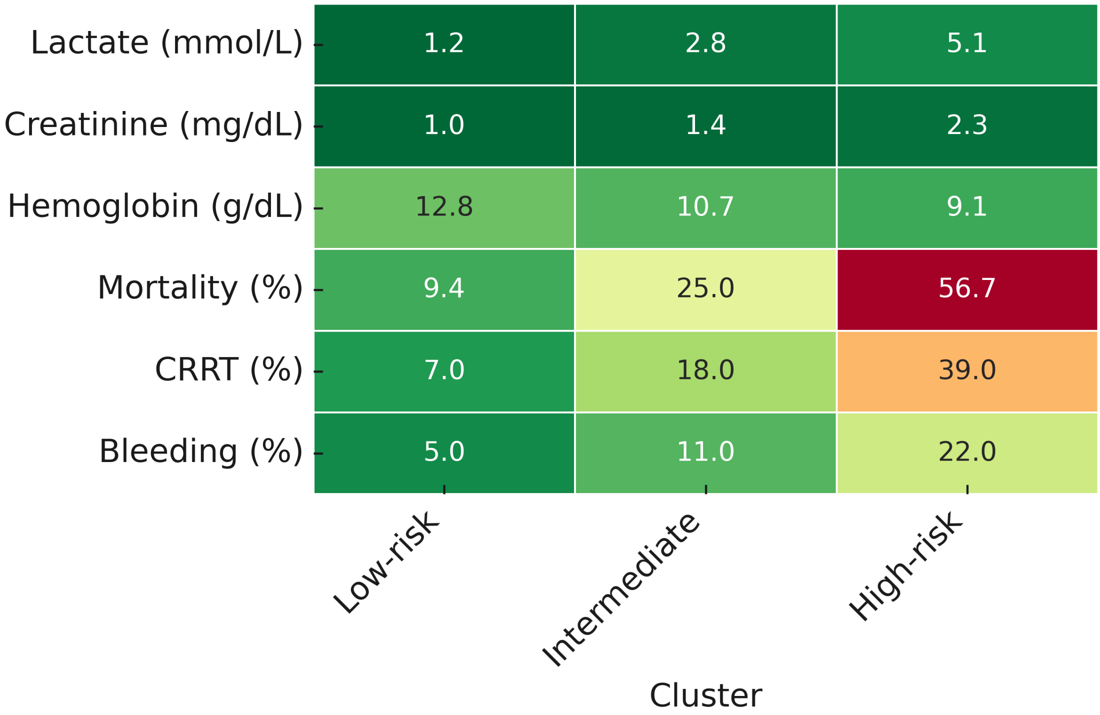
Lactate (mmol/L)
Creatinine (mg/dL)
Hemoglobin (g/dL)
Mortality (%)
CRRT (%)
Bleeding (%)
1.2
2.8
5.1
1.0
1.4
2.3
12.8
10.7
9.1
9.4
25.0
56.7
7.0
18.0
39.0
5.0
11.0
22.0
Low-risk
Intermediate
High-risk
Cluster
