# Supplementary material for: Evaluation of dual-lumen pulmonary artery cannulation in extracorporeal right ventricular support
Source: JTCVS Open. 2026 Mar 4;30:101699. doi: 10.1016/j.xjon.2026.101699 (PMC13131193; doi:10.1016/j.xjon.2026.101699)
Supplement: Online Data Supplement [file mmc16.docx]

**Original Research Article**

**Understanding how dual-lumen cannula for pulmonary artery cannulation reduces mortality and complications in dedicated extracorporeal right ventricular support: a comprehensive causal mediation and clustering analysis.**

**Supplementary Material**

**PLACE Investigators**

Jos Maessen, MD, PhD, Department of Cardio-Thoracic Surgery, Heart and Vascular Centre, Maastricht University Medical Centre, Maastricht, the Netherlands; Cardiovascular Research Institute Maastricht (CARIM), Maastricht, The Netherlands;

Opema Lohese, BS, Department of Cardiology, Minneapolis Heart Center, Minneapolis, US;

Davide Pacini, MD, PhD, Department of Cardiac Surgery, Sant’ Orsola University Hospital, Bologna, Italy;

Sofia Martin Suarez, MD, PhD, Department of Cardiac Surgery, Sant’ Orsola University Hospital, Bologna, Italy;

Luca Botta, MD, PhD, Department of Cardiac Surgery, Sant’ Orsola University Hospital, Bologna, Italy;

Daniel Zimpfer, MD, Department of Cardiac Surgery, Medical University of Vienna, Vienna, Austria;

Anne-Kristin Schaefer, MD, Department of Cardiac Surgery, Medical University of Vienna, Vienna, Austria;

Philipp Szalkiewicz, MD, Department of Cardiac Surgery, Medical University of Vienna, Vienna, Austria;

Bart Meyns, MD, PhD, Department of Cardiac Surgery, University Hospitals Leuven, Leuven, Belgium;

Marie De Vos, MD, Department of Cardiac Surgery, University Hospitals Leuven, Leuven, Belgium;

Leen Vercaemst, BSc, Department of Cardiac Surgery, University Hospitals Leuven, Leuven, Belgium;

Matthias Karck, MD, Department of Cardiac Surgery, University Hospital Heidelberg, Heidelberg, Germany;

Anna L Meyer, MD, Department of Cardiac Surgery, University Hospital Heidelberg, Heidelberg, Germany;

Ana J Holler, MD, Department of Cardiac Surgery, University Hospital Heidelberg, Heidelberg, Germany;

Matthias Jacquet-Lagrèze, MD, PhD, Department of Anesthesia and Intensive Care Unit, Louis Pradel Cardiologic Hospital, Lyon, France;

Jean Francois Obadia, MD, Department of Cardiac Surgery, Louis Pradel Cardiologic Hospital, Lyon, France;

Jean Luc Fellahi, , MD, PhD, Department of Anesthesia and Intensive Care Unit, Louis Pradel Cardiologic Hospital, Lyon, France;

Amy S Wang, BS, Department of Cardiac Surgery, Columbia University Irving Medical Center, New York, US;

Nikolaos Kalampokas, MD, Department of Cardiac Surgery, University Hospital Düsseldorf, Düsseldorf, Germany;

Artur Lichtenberg, MD, PhD, Department of Cardiac Surgery, University Hospital Düsseldorf, Düsseldorf, Germany;

Hug Aubin, MD, Department of Cardiac Surgery, University Hospital Düsseldorf, Düsseldorf, Germany;

Chih-Hsien Wang, MD, PhD, Department of Cardiovascular Surgery, National Taiwan University Hospital, Taipei City, Taiwan;

Chun-Cheng Huang, MD, Department of Cardiovascular Surgery, National Taiwan University Hospital, Taipei City, Taiwan;

Heng-Wen Chou, MD, Department of Cardiovascular Surgery, National Taiwan University Hospital, Taipei City, Taiwan;

Severino Iesu, MD, Emergency Cardiac Surgery - Cardio-Thoracic-Vascular Department, University Hospital San Giovanni di Dio e Ruggi D'Aragona, Salerno, Italy;

Generoso Mastrogiovanni, MD, Emergency Cardiac Surgery - Cardio-Thoracic-Vascular Department, University Hospital San Giovanni di Dio e Ruggi D'Aragona, Salerno, Italy;

Vittoria Iennaco, MD, Emergency Cardiac Surgery - Cardio-Thoracic-Vascular Department, University Hospital San Giovanni di Dio e Ruggi D'Aragona, Salerno, Italy;

Victoria M Taylor, MBChB, Department of Intensive Care Unit, Guy's and St Thomas' NHS Foundation Trust, London, UK;

Nicholas A Barret, MBBS, Department of Intensive Care Unit, Guy's and St Thomas' NHS Foundation Trust, London, UK; Centre for Human & Applied Physiological Sciences, Faculty of Life Sciences & Medicine, King’s College London, London, UK;

Carlos V Elzo Kraemer, MD, Department of Intensive Care, Leiden University Medical Center, Leiden, the Netherlands;

Jorge E Lopez Matta, MD, Department of Intensive Care, Leiden University Medical Center, Leiden, the Netherlands;

Jeroen J Janson, MD, Department of Intensive Care, Leiden University Medical Center, Leiden, the Netherlands;

Camilla L'Acqua, MD, Department of Intensive Care Unit, IRCCS Centro Cardiologico Monzino, Milano, Italy;

Fabiana L Rossi, BSc, PhD, Department of Cardiac Surgery, IRCCS Centro Cardiologico Monzino, Milano, Italy;

Antonella Bertera, BSc, Department of Cardiac Surgery, IRCCS Centro Cardiologico Monzino, Milano, Italy;

Giulia Maj, MD, Department of Cardiothoracic and Vascular Anesthesia and Intensive Care, SS Antonio e Biagio e Cesare Arrigo Hospital, Alessandria, Italy;

Andrea Audo, MD, Department of Cardiac Surgery, SS Antonio e Biagio e Cesare Arrigo Hospital, Alessandria, Italy;

Stephanie Bertolin, MD, Department of Cardiothoracic and Vascular Anesthesia and Intensive Care, SS Antonio e Biagio e Cesare Arrigo Hospital, Alessandria, Italy.

**Supplementary Table S1. STROBE Checklist**

| **Section and Item** | **Item No.** | **Recommendation** | **Reported in Section/Page** |
| --- | --- | --- | --- |
| Title and abstract | 1 | Indicate the study’s design with a commonly used term in the title or the abstract. Provide in the abstract an informative and balanced summary of what was done and what was found. | Title page, Abstract |
| Introduction | 2 | Explain the scientific background and rationale for the investigation being reported. | Introduction |
| Objectives | 3 | State specific objectives, including any prespecified hypotheses. | Introduction |
| Study design | 4 | Present key elements of study design early in the paper. | Methods |
| Setting | 5 | Describe the setting, locations, and relevant dates, including periods of recruitment, exposure, follow-up, and data collection. | Methods |
| Participants | 6 | Give the eligibility criteria, and the sources and methods of selection of participants. Describe methods of follow-up. | Methods |
| Variables | 7 | Clearly define all outcomes, exposures, predictors, potential confounders, and effect modifiers. Give diagnostic criteria, if applicable. | Methods |
| Data sources/measurement | 8 | For each variable of interest, give sources of data and details of methods of assessment (measurement). Describe comparability of assessment methods if there is more than one group. | Methods |
| Bias | 9 | Describe any efforts to address potential sources of bias. | Methods |
| Study size | 10 | Explain how the study size was arrived at. | Methods |
| Quantitative variables | 11 | Explain how quantitative variables were handled in the analyses. If applicable, describe which groupings were chosen and why. | Methods |
| Statistical methods | 12 | Describe all statistical methods, including those used to control for confounding. Describe any methods used to examine subgroups and interactions. Explain how missing data were addressed. Describe any sensitivity analyses. | Methods |
| Participants | 13 | Report numbers of individuals at each stage of study—e.g., numbers potentially eligible, examined for eligibility, confirmed eligible, included in the study, completing follow-up, and analyzed. | Results |
| Descriptive data | 14 | Give characteristics of study participants (e.g., demographic, clinical, social) and information on exposures and potential confounders. | Results |
| Outcome data | 15 | Report numbers of outcome events or summary measures over time. | Results |
| Main results | 16 | Give unadjusted estimates and, if applicable, confounder-adjusted estimates and their precision (e.g., 95% CI). Make clear which confounders were adjusted for and why they were included. | Results |
| Other analyses | 17 | Report other analyses done—e.g., analyses of subgroups and interactions, and sensitivity analyses. | Results |
| Key results | 18 | Summarize key results with reference to study objectives. | Discussion |
| Limitations | 19 | Discuss limitations of the study, taking into account sources of potential bias or imprecision. | Discussion |
| Interpretation | 20 | Give a cautious overall interpretation of results considering objectives, limitations, multiplicity of analyses, results from similar studies, and other relevant evidence. | Discussion |
| Generalisability | 21 | Discuss the generalisability (external validity) of the study results. | Discussion |
| Funding | 22 | Give the source of funding and the role of the funders for the present study and, if applicable, for the original study on which the present article is based. | Disclosure |

**Supplementary Table S2. RVAD Cannulation Strategies and Configurations**

| **Cannula Placement** | **Technique / Access Route** |
| --- | --- |
| Inflow – Surgical | Direct cannulation of right atrium |
| Inflow – Surgical | Right atrial cannulation via femoral venous conduit |
| Inflow – Percutaneous | Femoral or jugular venous access to right atrium |
| Outflow – Surgical | Direct pulmonary artery cannulation |
| Outflow – Surgical | Pulmonary artery graft access (chimney technique) |
| Outflow – Percutaneous | Right jugular double-lumen cannula |
| Outflow – Percutaneous | Single-lumen cannula through jugular or femoral route |

**Supplementary Table S3. Distribution of primary and secondary outcomes in extracorporeal life support (ECLS) for refractory right ventricular failure stratified by dual-lumen (DL) and single-lumen (SL) cannulation.**

| **Outcome** | **Gruppo** | **N** | **Evento sì**  **(n)** | **% Evento sì** | **Mediana (giorni)** | **IQR (Q1–Q3)** |
| --- | --- | --- | --- | --- | --- | --- |
| In-hospital mortality | SL | 183 | 73 | 39.9% | — | — |
| In-hospital mortality | DL | 162 | 70 | 43.2% | — | — |
| 30-day mortality | SL | 183 | 59 | 32.2% | — | — |
| 30-day mortality | DL | 162 | 51 | 31.5% | — | — |
| Bleeding | SL | 172 | 59 | 34.3% | — | — |
| Bleeding | DL | 134 | 32 | 23.9% | — | — |
| CRRT requirement | SL | 174 | 45 | 25.9% | — | — |
| CRRT requirement | DL | 134 | 29 | 21.6% | — | — |
| Thromboembolic Events | SL | 171 | 22 | 12.9% | — | — |
| Thromboembolic Events | DL | 132 | 15 | 11.4% | — | — |
| ICU Length of Stay | SL | 174 | — | — | 30.0 | 16.5 – 43.5 |
| ICU Length of Stay | DL | 134 | — | — | 21.0 | 12.3 – 40.5 |
| Hospital Length of Stay | SL | 174 | — | — | 42.0 | 26.5 – 62.5 |
| Hospital Length of Stay | DL | 134 | — | — | 41.5 | 24.3 – 73.3 |

CRRT, continuous renal replacement therapy; ICU, intensive care unit; IQR, interquartile range; DL, dual-lumen cannulation; SL, single-lumen cannulation.
Percentages are calculated based on the number of patients with available data in each group.
Data are unweighted.
Bleeding events defined as those requiring transfusion or surgical intervention.
Thromboembolic events include clinically diagnosed deep vein thrombosis, pulmonary embolism, or stroke.

**Supplementary Table S4. Unweighted patient characteristics**

| **Pre-Implant Features** | **Single-Lumen**  **N=166** | | | | **Dual-Lumen**  **N=179** | | | **p** | |  |  |
| --- | --- | --- | --- | --- | --- | --- | --- | --- | --- | --- | --- |
|  | **Nr.** | | **%** | | **Nr.** | **%** | |  |  |  |  |
| Gender |  | |  | |  |  | | 0.625 | |  |  |
| Male | 128 | | 77.1 | | 134 | 74.9 | |  | |  |  |
| Female | 38 | | 22.9 | | 45 | 25.1 | |  | |  |  |
| Age (median, IQR) | 58 (48-68) | | | | 60 (50.69) | | | 0.040 | |  |  |
| <50 | 50 | | 30.1 | | 51 | 28.5 | |  | |  |  |
| 50-60 | 45 | | 27.1 | | 40 | 22.3 | |  | |  |  |
| >60 | 71 | | 42.8 | | 88 | 49.2 | |  | |  |  |
| BMI (median, IQR) | 25.8 (23.6-29.7) | | | | 28.1 (24.3-32.5) | | | <0.001 | |  |  |
| Weight |  | |  | |  |  | | 0.007 | |  |  |
| Normal Weight | 72 | | 43.4 | | 55 | 30.7 | |  | |  |  |
| Overweight | 54 | | 32.5 | | 56 | 31.8 | |  | |  |  |
| Mild Obesity | 26 | | 15.7 | | 39 | 21.2 | |  | |  |  |
| Moderate Obesity | 13 | | 7.8 | | 16 | 9.5 | |  | |  |  |
| Severe Obesity | 1 | | 0.6 | | 12 | 6.7 | |  | |  |  |
| Patient’s Condition at the Implant |  | |  | |  |  | | 0.031 | |  |  |
| Hemodynamic Unstable | 52 | | 31.3 | | 72 | 40.2 | | 0.085 | |  |  |
| Previous Cardiac Arrest | 14 | | 8.4 | | 24 | 13.4 | | 0.140 | |  |  |
| Profound Cardiogenic Shock | 100 | | 60.2 | | 83 | 46.4 | | 0.010 | |  |  |
| Diuresis Output at the Implant |  | |  | |  |  | | 0.079 | |  |  |
| Normal | 103 | | 62.0 | | 111 | 62.0 | |  | |  |  |
| Oliguric | 41 | | 24.7 | | 56 | 31.3 | |  | |  |  |
| Anuric | 22 | | 13.3 | | 12 | 6.7 | |  | |  |  |
| LVEF (%) (at echocardiography) | 25 (15-35) | | | 20 (10-30) | | | 0.004 | |  | |  |
| Comorbidities |  | | |  | | |  | |  | |  |
| Hypertension | 62 | 37.6 | | 114 | | 63.7 | <0.001 | |  | |  |
| Smoke | 43 | 25.9 | | 73 | | 40.8 | 0.003 | |  | |  |
| DM | 37 | 22.3 | | 55 | | 30.7 | 0.077 | |  | |  |
| Dyslipidaemia | 53 | 31.9 | | 75 | | 41.9 | 0.055 | |  | |  |
| COPD | 24 | 14.5 | | 24 | | 13.4 | 0.779 | |  | |  |
| Chronic Kidney Disease |  |  | |  | |  | 0.626 | |  | |  |
| No dialysis | 46 | 27.7 | | 57 | | 31.8 |  | |  | |  |
| Dialysis | 7 | 4.2 | | 9 | | 5.0 |  | |  | |  |
| Cerebrovascular Accident | 15 | 9.0 | | 9 | | 5.0 | 0.144 | |  | |  |
| Peripheral Vascular Disease | 7 | 4.2 | | 21 | | 11.8 | 0.029 | |  | |  |
| Pulmonary Hypertension | 67 | 44.4 | | 39 | | 21.8 | 0.001 | |  | |  |
| Atrial Fibrillation | 45 | 27.1 | | 56 | | 31.2 | 0.394 | |  | |  |
| PM/ICD | 43 | 25.9 | | 73 | | 39.7 | <0.001 | |  | |  |
| AMI | 48 | 28.9 | | 63 | | 35.2 | 0.212 | |  | |  |
| Previous PCI | 57 | 34.3 | | 59 | | 33.0 | 0.787 | |  | |  |
| Redo after Cardiac Operation | 33 | 19.9 | | 61 | | 34.1 | 0.010 | |  | |  |
| Chronic RVF | 67 | 40.4 | | 73 | | 40.8 | 0.937 | |  | |  |
| Chronic RVF: Etiologies |  |  | |  | |  |  | |  | |  |
| Right-side Valve Disease | 29 | 17.5 | | 11 | | 6.1 | 0.001 | |  | |  |
| Left-side Valve Disease | 12 | 7.2 | | 35 | | 19.6 | 0.001 | |  | |  |
| COPD | 0 | 0 | | 3 | | 1.7 | 0.249 | |  | |  |
| Post-AMI | 22 | 13.3 | | 15 | | 8.4 | 0.144 | |  | |  |
| Chronic Thromboembolism | 1 | 0.6 | | 1 | | 0.6 | 1.000 | |  | |  |
| Endocrine Crisis | 1 | 0.6 | | 2 | | 1.1 | 1.000 | |  | |  |
| Myocarditis | 1 | 0.6 | | 6 | | 3.4 | 0.123 | |  | |  |
| ARVD | 0 | 0 | | 2 | | 1.1 | 0.499 | |  | |  |
| Cor Pulmonale | 2 | 1.2 | | 0 | | 0 | 0.231 | |  | |  |
| ECLS-Pre RV Mechanical Support |  |  | |  | |  | 0.120 | |  | |  |
| VV | 1 | 0.6 | | 3 | | 1.7 | 0.624 | |  | |  |
| VA | 81 | 48.8 | | 69 | | 38.5 | 0.048 | |  | |  |
| RV Mechanical Support Indications |  |  | |  | |  |  | |  | |  |
| RVF | 14 | 8.4 | | 10 | | 5.6 | 0.299 | |  | |  |
| Failure Weaning CPB | 8 | 4.8 | | 5 | | 2.8 | 0.401 | |  | |  |
| Previous Cardiac Arrest | 14 | 8.4 | | 10 | | 5.6 | 0.397 | |  | |  |
| Cardiogenic Shock | 53 | 31.9 | | 48 | | 26.8 | 0.297 | |  | |  |
| BiV Failure | 10 | 6.0 | | 6 | | 3.4 | 0.308 | |  | |  |
| Respiratory Dysfunction | 3 | 1.8 | | 4 | | 2.2 | 1.000 | |  | |  |
| Intractable Arrhythmia | 5 | 3.0 | | 3 | | 1.7 | 0.410 | |  | |  |

Abbreviations : AMI, acute myocardial infarction; ARVD, arrhythmogenic right ventricular dysplasia; BiV, biventricular; BMI, body mass index; COPD, chronic obstructive pulmonary disease; CPB, cardiopulmonary bypass; DM, diabetes mellitus; ECLS, extracorporeal life support; IQR, interquartile range; LVEF, left ventricle ejection fraction; LVHD, left ventricular heart disease; PCI, percutaneous coronary intervention; PM/ICD, pacemaker/implantable cardioverter-defibrillator; Pt, patient; RV, right ventricle; RVF, right ventricular failure; VA, veno-arterial; VV, veno-venous.

**Supplementary Table S5. Unweighted Implant characteristics of the right ventricle support**

| **Implant Features** | **Single-Lumen**  **N=166** | | **Dual-Lumen**  **N=179** | | **p** |
| --- | --- | --- | --- | --- | --- |
|  | **Nr.** | **%** | **Nr.** | **%** |  |
| Approach |  |  |  |  | < 0.001 |
| Percutaneous | 31 | 18.7 | 179 | 100.0 |  |
| Surgical Graft Facilitated | 93 | 56.0 | 0 | 0 |  |
| Surgical Central | 42 | 25.3 | 0 | 0 |  |
| Place of Implant - Guide |  |  |  |  | < 0.001 |
| Cath Lab - Fluoroscopy Guide | 7 | 4.2 | 76 | 42.5 |  |
| Hybrid Room - Fluoroscopy Guide | 31 | 18.7 | 72 | 40.2 |  |
| Bedside - Echo Guide | 1 | 0.6 | 31 | 17.3 |  |
| Operating Room | 127 | 76.5 | 0 | 0 |  |

**Supplementary Table S6. Complications during right ventricle support (Unweighted Data)**

| **Complications** | **Single-Lumen**  **N=166** | | **Dual-Lumen**  **N=179** | | **P-Value** |  |
| --- | --- | --- | --- | --- | --- | --- |
|  |  |  |  |  |  |  |
|  | **Nr.** | **%** | **Nr.** | **%** |  |  |
| Complications | 162 | 97.6 | 132 | 73.7 | < 0.001 |  |
| Clots in PA Cannula | 22 | 13.3 | 11 | 6.1 | 0.025 |  |
| Pulmonary Valve Insufficiency | 0 | 0 | 1 | 0.6 | 0.335 |  |
| Perforation of RV | 0 | 0 | 2 | 1.1 | 0.172 |  |
| Cardiac Tamponade | 26 | 15.7 | 27 | 15.1 | 0.882 |  |
| Bleeding | 74 | 44.6 | 71 | 39.7 | 0.356 |  |
| Surgical-Related | 50 | 30.1 | 57 | 31.8 | 0.730 |  |
| Cannula Site-Related | 11 | 6.6 | 2 | 1.1 | 0.007 |  |
| Lung-Related | 19 | 11.4 | 12 | 6.7 | 0.124 |  |
| Coagulation-Related | 7 | 4.2 | 0 | 0 | 0.006 |  |
| Thrombo-Embolic Events | 8 | 4.8 | 11 | 6.1 | 0.590 |  |
| Vascular | 12 | 7.2 | 12 | 6.7 | 0.848 |  |
| Infection | 94 | 56.6 | 68 | 38.0 | 0.001 |  |
| Neurological | 34 | 20.5 | 20 | 11.2 | 0.017 |  |
| Brain Edema | 9 | 5.4 | 4 | 2.2 | 0.120 |  |
| Cerebral Hemorrhage | 10 | 6.0 | 6 | 3.4 | 0.238 |  |
| Seizure | 7 | 4.2 | 3 | 1.7 | 0.160 |  |
| Stroke | 14 | 8.4 | 7 | 3.9 | 0.079 |  |
| Vasospasm | 3 | 1.8 | 0 | 0 | 0.071 |  |
| Not possible Waking Up | 5 | 3.0 | 0 | 0 | 0.019 |  |
| Polyneuropathy | 4 | 2.4 | 0 | 0 | 0.037 |  |
| Liver Failure | 42 | 25.3 | 28 | 15.6 | 0.026 |  |
| Renal Failure | 120 | 72.3 | 116 | 64.8 | 0.135 |  |
| CVVH | 95 | 57.2 | 96 | 53.6 | 0.502 |  |
| Respiratory | 70 | 42.2 | 71 | 39.7 | 0.636 |  |
| VAP | 46 | 27.7 | 56 | 31.3 | 0.467 |  |
| VILI | - | - | 3 | 1.7 | 0.094 |  |
| Lung Hemorrhage | 20 | 12.0 | 12 | 6.7 | 0.087 |  |
| Respiratory Insufficiency | 4 | 2.4 | 1 | 0.6 | 0.151 |  |
| Hospital-acquired Pneumonia | 2 | 1.2 | 1 | 0.6 | 0.518 |  |
| ARDS | 5 | 3.0 | - | - | 0.019 |  |
| Gastro-intestinal | 27 | 16.3 | 32 | 17.9 | 0.691 |  |
| Ischemic | 13 | 7.8 | 22 | 12.3 | 0.170 |  |
| Haemorrhagic | 14 | 8.4 | 10 | 5.6 | 0.299 |  |
| Arrhythmia | 73 | 44.0 | 71 | 39.7 | 0.417 |  |
|  |  |  |  |  |  |  |

**Abbreviations.** VAP: Ventilator-Associated Pneumonia; VILI: Ventilator-Induced Lung Injury; ARDS: Acute Respiratory Distress Syndrome.

**Supplementary Table S7. Outcome associated with pulmonary artery cannulation with single-lumen or with dual-lumen cannula (Unweighted Data)**

| **Outcome** | **Single-Lumen**  **N=166** | | **Dual-Lumen**  **N=179** | | **p** |
| --- | --- | --- | --- | --- | --- |
|  |  |  |  |  |  |
|  | **Nr.** | **%** | **Nr.** | **%** |  |
| Death on RV Support | 41 | 24.7 | 49 | 27.4 | 0.572 |
| MOF | 24 | 14.5 | 26 | 14.5 | 0.603 |
| Sepsis | 3 | 1.8 | 8 | 4.5 | 0.194 |
| Cardiac Arrest | 0 | 0 | 6 | 3.4 | 0.031 |
| Vasoplegia | 6 | 3.6 | 1 | 0.6 | 0.044 |
| Bleeding | 6 | 3.6 | 3 | 1.7 | 0.180 |
| Neurological death | 5 | 3.0 | 2 | 1.1 | 0.152 |
| Bowel Ischemia | 3 | 1.8 | 2 | 1.1 | 0.505 |
| Low Output Syndrome | 5 | 3.0 | 5 | 2.8 | 0.765 |
| Fluid Overload | 1 | 0.6 | 0 | 0 | 0.272 |
| Withdrawal | 4 | 2.4 | 2 | 1.1 | 0.282 |
| Weaning | 125 | 75.3 | 130 | 72.6 | 0.624 |
| Death after Weaning | 32 | 19.3 | 18 | 10.1 | 0.015 |
| Causes of Death after  Weaning |  |  |  |  |  |
| MOF | 12 | 7.2 | 8 | 4.5 | 0.818 |
| Sepsis | 12 | 7.2 | 3 | 1.7 | 0.033 |
| Cardiac Arrest | 1 | 0.6 | 0 | 0 | 0.391 |
| Vasoplegia | 2 | 1.2 | 0 | 0 | 0.219 |
| Bleeding | 1 | 0.6 | 2 | 1.1 | 0.367 |
| Neurological death | 3 | 1.8 | 2 | 1.1 | 0.929 |
| Bowel Ischemia | 1 | 0.6 | 0 | 0 | 0.391 |
| Low Output Syndrome | 2 | 1.2 | 2 | 1.1 | 0.729 |
| Fluid Overload | 0 | 0 | 1 | 0.6 | 0.233 |
| Long-Term RVAD | 4 | 2.4 | 39 | 21.8 | < 0.001 |
| HTx | 14 | 8.4 | 18 | 10.1 | 0.604 |
| Alive at Hospital Discharge | 93 | 56.0 | 112 | 62.6 | 0.216 |
| Alive at 3 Months Follow-up | 90 | 53.9 | 106 | 59.0 | 0.346 |
|  |  |  |  |  |  |

Abbreviations. MOF. multiorgan failure; RV, right ventricle; RVAD, right ventricular assist device.

**Supplementary Table S8. Differences Before and After Weighting for Propensity Score Covariates**

| **Variable** | **ASMD Before Weighting** | **ASMD After Weighting** | **Absolute Difference** |
| --- | --- | --- | --- |
| Age | 0.222 | 0.156 | 0.066 |
| Sex | 0.052 | 0.022 | 0.031 |
| BMI | 0.389 | 0.166 | 0.123 |
| Hypertension | 0.544 | 0.055 | 0.489 |
| Diabetes Mellitus | 0.192 | 0.036 | 0.155 |
| COPD | 0.030 | 0.059 | 0.029 |
| Atrial Fibrillation | 0.092 | 0.073 | 0.018 |
| Chronic Kidney Disease | 0.100 | 0.021 | 0.121 |
| Pulmonary Hypertension | 0.417 | 0.133 | 0.416 |
| Peripheral Vascular Disease | 0.254 | 0.145 | 0.09 |
| CVA | 0.158 | 0.102 | 0.056 |
| Previous PCI | 0.029 | 0.081 | 0.052 |
| Redo of Cardiac Surgery | 0.332 | 0.072 | 0.26 |
| LVEF | 0.661 | 0.166 | 0.395 |
| MAP | 0.455 | 0.111 | 0.244 |
| CVP | 0.485 | 0.174 | 0.174 |
| PaO₂ | 0.196 | 0.041 | 0.645 |
| Platelets | 0.218 | 0.119 | 0.101 |
| Bilirubin | 0.256 | 0.159 | 0.203 |
| Creatinine | 0.068 | 0.043 | 0.025 |
| Lactate | 0.452 | 0.163 | 0.111 |
| Smoking History | 0.319 | 0.136 | 0.017 |
| Dyslipidemia | 0.207 | 0.164 | 0.056 |

# **Supplementary Table S9. Univariable IPTW Analysis – Primary and Secondary Outcomes**

| **Outcome** | **Measure** | **Estimate (95% CI)** | **p** |
| --- | --- | --- | --- |
| In-Hospital Mortality | wOR | 1.15 (0.75–1.76) | 0.5323 |
| 30-Day Mortality | wOR | 0.97 (0.61–1.52) | 0.88 |
| Thromboembolic Events | wOR | 1.06 (0.49–2.27) | 0.8818 |
| Bleeding Events | wOR | 1.21 (0.79–1.85) | 0.3926 |
| Bleeding or Thromboembolism | wOR | 1.32 (0.86–2.01) | 0.2046 |
| CRRT Requirement | wOR | 1.06 (0.70–1.63) | 0.7757 |
| Successful Weaning from RV Support | wOR | 1.07 (0.66–1.72) | 0.7826 |
| 3-Month Mortality | wOR | 1.08 (0.70–1.67) | 0.7128 |
| ICU Length of Stay (days) | Δ Mean | -2.44 (-9.41–+4.52) | 0.4904 |
| Hospital Length of Stay (days) | Δ Mean | +1.54 (-8.97–+12.04) | 0.7738 |

Abbreviations DL = Dual-lumen; SL = Single-lumen; IPTW = Inverse Probability of Treatment Weighting;
wOR = weighted Odds Ratio; wHR = weighted Hazard Ratio; Δ Mean = difference in weighted mean values between groups; CI = Confidence Interval; CRRT = Continuous Renal Replacement Therapy; ICU = Intensive Care Unit. Note; wOR and wHR values represent the relative likelihood or risk over time for binary outcomes after applying IPTW adjustment. Δ Mean refers to the adjusted difference in continuous outcome means between DL and SL groups.

# **Supplementary Table S10. Multivariable IPTW Analysis – Primary and Secondary Outcomes**

| **Outcome** | **Measure** | **Estimate (95% CI)** | **p-value** | **Interpretation** |
| --- | --- | --- | --- | --- |
| In-Hospital Mortality | wOR | 1.15 (0.75–1.76) | 0.5323 | No significant difference |
| 30-Day Mortality | wOR | 0.97 (0.61–1.52) | 0.88 | No significant difference |
| Thromboembolic Events | wOR | 1.06 (0.49–2.27) | 0.8818 | No significant difference |
| Bleeding Events | wOR | 0.50 (0.32–0.77) | **0.0017** | **↓ Risk with DL** |
| Bleeding or Thromboembolism | wOR | 0.57 (0.39–0.84) | **0.004** | **↓ Composite risk with DL** |
| CRRT Requirement | wOR | 0.59 (0.37–0.92) | **0.0194** | **↓ Risk with DL** |
| Successful Weaning from RV Support | wOR | 1.07 (0.66–1.72) | 0.7826 | No significant difference |
| 3-Month Mortality | wOR | 1.08 (0.70–1.67) | 0.7128 | No significant difference |
| ICU Length of Stay (days) | Δ Mean | -2.44 (-9.41–+4.52) | 0.4904 | No significant difference |
| Hospital Length of Stay (days) | Δ Mean | +1.54 (-8.97–+12.04) | 0.7738 | No significant difference |

Abbreviations: DL = Dual-lumen; SL = Single-lumen; IPTW = Inverse Probability of Treatment Weighting; wOR = weighted Odds Ratio; Δ Mean = difference in weighted mean values between groups; CI = Confidence Interval; CRRT = Continuous Renal Replacement Therapy; ICU = Intensive Care Unit.
Note: Interpretations are based on statistical significance (p < 0.05) and direction of effect. Note: wOR and wHR values represent the relative likelihood or risk over time for binary outcomes after applying IPTW adjustment. Δ Mean refers to the adjusted difference in continuous outcome means between DL and SL groups. Interpretations are based on statistical significance (p < 0.05) and direction of effect.

| **Outcome** | **Interaction Term** | **Adjusted OR** | **95% CI** | **p** | **Interpretation** |
| --- | --- | --- | --- | --- | --- |
| 30-day mortality | DL * PaO₂ (PaO2pre) | 0.99 | 0.99–1.00 | **0.007** | **↓ Mortality with DL in hypoxemia** |
| In-hospital mortality | DL * PaO₂ (PaO2pre) | 0.99 | 0.99–1.00 | **0.01** | **↓ Mortality with DL in hypoxemia** |
| CRRT requirement | DL * Creatinine ≥1.5 | 0.29 | 0.11–0.76 | **0.01** | **↓ CRRT with DL in renal impairment** |
| Bleeding | DL * Platelets <150 | 1.17 | 0.48–2.84 | 0.72 | No significant interaction |
| In-hospital mortality | DL * Cannulation site | 1.04 | 0.64-1.70 | 0.87 | No significant interaction |
| 30-day mortality | DL * Cannulation site | 0.96 | 0.59-155 |  | No significant interaction |
| In-hospital mortality | DL * Oxygenator use | 1.08 | \| 0.65–1.78 \| \| --- \| | \| 0.74 \| \| --- \| | No significant interaction |
| 30-day mortality | DL * Oxygenator use | 1.02 | 0.59-1.75 | 0.91 | No significant interaction |
|  |  |  |  |  |  |

**Supplementary Table S11. Interaction Analysis**

Abbreviations: DL = Dual-lumen cannula; SL = Single-lumen cannula; PaO₂ = Partial pressure of arterial oxygen; OR = Odds Ratio; CRRT = Continuous Renal Replacement Therapy; CI = Confidence Interval.

# **Supplementary Table S12. Full results of causal mediation analyses**

| **Outcome** | **Mediator** | **ACME** | **ADE** | **% Mediated** | **p (ACME)** |
| --- | --- | --- | --- | --- | --- |
| In-hospital Mortality | HBpost | -0.022 | 0.142 | -16.2% | 0.056 |
| 30-day Mortality | WBCpost | 0.054 | 0.020 | 87.8% | 0.38 |
| Bleeding | BilTotpost | 0.029 | 0.093 | 22.8% | 0.28 |
| CRRT Requirement | Lactatepost | 0.032 | 0.076 | 29.8% | 0.41 |
| In-hospital Mortality | Creatininpost | 0.006 | 0.114 | 4.0% | 0.46 |
| 30-day Mortality | ALTpost | 0.058 | 0.057 | 51.5% | 0.15 |
| Bleeding | Sepsis_A | 0.004 | 0.107 | 0.8% | 0.96 |
| CRRT Requirement | Arrhythmia | 0.008 | 0.113 | 6.3% | 0.39 |
| In-hospital Mortality | HBpost | -0.018 | 0.128 | -12.5% | 0.074 |
| 30-day Mortality | WBCpost | 0.047 | 0.027 | 63.5% | 0.41 |
| Bleeding | BilTotpost | 0.026 | 0.099 | 20.9% | 0.32 |
| CRRT Requirement | Lactatepost | 0.030 | 0.078 | 27.7% | 0.47 |
| In-hospital Mortality | Creatininpost | 0.005 | 0.116 | 4.3% | 0.50 |
| 30-day Mortality | ALTpost | 0.053 | 0.059 | 47.4% | 0.16 |
| Bleeding | Sepsis_A | 0.004 | 0.109 | 0.9% | 0.94 |
| CRRT Requirement | Arrhythmia | 0.006 | 0.115 | 4.9% | 0.47 |
| In-hospital Mortality | HBpost | 0.015 | 0.084 | 15.1% | 0.24 |
| 30-day Mortality | WBCpost | 0.029 | 0.070 | 29.2% | 0.27 |
| Bleeding | BilTotpost | 0.011 | 0.088 | 10.9% | 0.33 |
| CRRT Requirement | Lactatepost | 0.022 | 0.077 | 22.2% | 0.26 |
| In-hospital Mortality | Creatininpost | 0.008 | 0.091 | 8.1% | 0.24 |
| 30-day Mortality | ALTpost | 0.033 | 0.066 | 33.4% | 0.14 |
| Bleeding | Sepsis_A | 0.005 | 0.094 | 5.4% | 0.48 |
| CRRT Requirement | Arrhythmia | 0.009 | 0.090 | 9.3% | 0.19 |
| In-hospital Mortality | HBpost | -0.005 | 0.065 | -7.1% | 0.51 |
| 30-day Mortality | WBCpost | 0.011 | 0.050 | 18.0% | 0.34 |
| Bleeding | BilTotpost | 0.007 | 0.054 | 11.4% | 0.39 |
| CRRT Requirement | Lactatepost | 0.010 | 0.051 | 16.4% | 0.33 |
| In-hospital Mortality | Creatininpost | 0.002 | 0.059 | 3.4% | 0.46 |
| 30-day Mortality | ALTpost | 0.011 | 0.050 | 18.7% | 0.34 |
| Bleeding | Sepsis_A | 0.000 | 0.061 | 0.2% | 0.95 |
| CRRT Requirement | Arrhythmia | 0.001 | 0.060 | 0.9% | 0.50 |

This table reports the estimated Average Causal Mediation Effects (ACME), Average Direct Effects (ADE), and percentage of the total effect mediated for each candidate postoperative mediator. None of the indirect effects (ACME) reached statistical significance (all p ≥ 0.05), but estimates are reported for completeness. All models are weighted using IPTW adjustment.

Abbreviations: DL = dual-lumen; SL = single-lumen; ACME = Average Causal Mediation Effect; ADE = Average Direct Effect; IPTW = Inverse Probability of Treatment Weighting; HBpost = postoperative hemoglobin; WBCpost = postoperative white blood cell count; BilTotpost = total bilirubin post-op; ALTpost = alanine aminotransferase post-op; Sepsis_A = postoperative sepsis; % Mediated = proportion of total effect mediated.

# **Supplementary Table S13. Clinical characteristics and outcomes by biochemical cluster**

| **Cluster** | **N (patients)** | **Biomarker Profile** | **Hospital Mortality (%)** | **CRRT (%)** | **Bleeding (%)** | **ICU LOS (days)** | **Hospital LOS (days)** |
| --- | --- | --- | --- | --- | --- | --- | --- |
| **1 (Low-risk)** | 80 | Low lactate, normal creatinine, high hemoglobin | 9.4% | 7% | 5% | 5.1 | 11.8 |
| **2 (Intermediate)** | 60 | Intermediate values | 25% | 18% | 11% | 6.5 | 14.3 |
| **3 (High-risk)** | 40 | High lactate, high creatinine, low hemoglobin | 56.7% | 39% | 22% | 11.2 | 21.5 |

Summary of the three patient clusters identified through k-means analysis using key postoperative biochemical markers. Each cluster demonstrates distinct profiles and significantly different clinical outcomes. ICU and hospital length of stay are reported as means (in days). Mortality and complication rates are expressed as percentages within each cluster. Clusters were derived via unsupervised k-means analysis using postoperative levels of lactate, creatinine, and hemoglobin.

Abbreviations: DL = dual-lumen; CRRT = continuous renal replacement therapy; LOS = length of stay; ICU = intensive care unit.

**Supplementary Statistical Analysis**

***Introduction and Objectives***

This study is a retrospective, multicenter observational cohort investigation designed to evaluate the clinical impact of double-lumen (DL) versus single-lumen (SL) cannulation in patients undergoing pulmonary artery cannulation in dedicated extracorporeal life support (ECLS) for refractory right ventricular failure (RVF) Data were collected from multiple tertiary centers to ensure the external validity and generalizability of the findings.

The primary objective of the statistical analysis was to rigorously estimate the effect of cannula type (DL vs. SL) on key clinical outcomes, including in-hospital mortality and 30-day mortality, while adequately controlling for potential confounding variables inherent in observational designs. Secondary objectives included the assessment of intermediate clinical endpoints, such as bleeding complications and continuous renal replacement therapy (CRRT) requirement, and the exploration of causal pathways through mediation analysis. In addition, interaction effects between cannula type and relevant clinical covariates were systematically evaluated to detect effect modification.

A fundamental challenge in observational research is the control of confounding bias arising from non-random treatment assignment. To mitigate this, propensity score methods with inverse probability of treatment weighting (IPTW) were employed, ensuring balance of measured covariates between treatment groups and enabling unbiased estimation of treatment effects.

***Description of Variables***

*Baseline Clinical Variables and Outcomes*

Baseline covariates included demographic parameters, clinical severity scores, and pre-cannulation laboratory and hemodynamic variables, comprehensively capturing patient clinical status prior to ECLS initiation. The primary outcomes of interest were all-cause in-hospital mortality and mortality at 30 days post-cannulation, selected for their clinical relevance and widespread acceptance as endpoints in critical care research.

Secondary outcomes encompassed major complications potentially influenced by cannula type, including bleeding events, thromboembolic complications, CRRT requirement, successful weaning from right ventricular support, and intensive care unit (ICU) and hospital length of stay. Proportion of Missing Data in Essential Clinical and Mediator Variables is shown in Supplementary **Table S14** .

*Mediator Variables: Postoperative Biomarkers*

A subset of postoperative laboratory parameters, serving as putative mediators in the causal pathway linking cannula type to clinical outcomes, were meticulously recorded. These included hemoglobin (HB), white blood cell count (WBC), total bilirubin (BilTot), lactate (Lactate), creatinine (Creatinin), alanine aminotransferase (ALT), the presence of postoperative sepsis (Sepsis_A), and occurrence of arrhythmias (Arrhythmia). These biomarkers were selected based on their pathophysiological relevance to organ function and systemic inflammatory response, providing mechanistic insights into how cannula choice may influence patient trajectory.

*Proportion of Missing Data in Essential Clinical and Mediator Variables*

A subset of postoperative laboratory parameters, serving as putative mediators in the causal pathway linking cannula type to clinical outcomes, were meticulously recorded. These included hemoglobin (HB), white blood cell count (WBC), total bilirubin (BilTot), lactate (Lactate), creatinine (Creatinin), alanine aminotransferase (ALT), the presence of postoperative sepsis (Sepsis_A), and occurrence of arrhythmias (Arrhythmia). These biomarkers were selected based on their pathophysiological relevance to organ function and systemic inflammatory response, providing mechanistic insights into how cannula choice may influence patient trajectory;

**Supplementary Table S14. Proportion of Missing Data in Essential Clinical and Mediator Variables**

| **Variable** | **Description** | **Missing Data (%)** |
| --- | --- | --- |
| Age | Patient age at ECMO initiation | 0.0% |
| Sex | Patient sex | 0.0% |
| PaO₂ pre-cannulation | Arterial oxygen pressure pre | 4.5% |
| Creatinine pre-op | Creatinine pre-cannulation | 3.8% |
| Hemoglobin post-op | Postoperative hemoglobin | 7.9% |
| WBC post-op | Postoperative white blood cells | 6.5% |
| Bilirubin post-op | Postoperative total bilirubin | 9.2% |
| Lactate post-op | Postoperative lactate | 8.7% |
| ALT post-op | Postoperative alanine aminotransferase | 10.5% |
| Sepsis post-op | Postoperative sepsis (binary) | 2.4% |
| Arrhythmia post-op | Postoperative arrhythmia (binary) | 1.7% |
| In-hospital mortality | Primary outcome | 0.0% |
| 30-day mortality | Secondary outcome | 0.0% |

***Handling of Missing Data***

*Exploratory Analysis of Missing Data*

An initial exploratory assessment was conducted to quantify the extent and pattern of missing data across all relevant variables, including baseline covariates used for propensity score estimation, mediator variables, and primary and secondary outcomes. The overall proportion of missingness was low to moderate. Specifically, the majority of key pre-cannulation variables and postoperative mediators exhibited missing data rates below the commonly accepted threshold of 10%, ensuring robust analytical validity. A limited number of secondary variables presented higher missingness rates, but these were deemed appropriate for statistical imputation without compromising the dataset’s integrity.

Missing data mechanisms were preliminarily evaluated to assess the plausibility of missing completely at random (MCAR), missing at random (MAR), or missing not at random (MNAR) assumptions. Formally:

- Missing Completely at Random (MCAR):


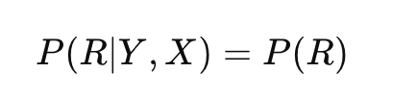


where R is the missingness indicator, Y is the data, and X are covariates; the probability of missingness is independent of observed and unobserved data.

- Missing at Random (MAR):


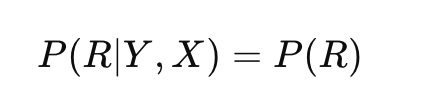


meaning missingness depends only on observed data X, but not on the missing values themselves.

- Missing Not at Random (MNAR):

Missingness depends on unobserved data values, violating MCAR and MAR assumptions.

Since MCAR was unlikely, and MAR plausible, Multiple Imputation techniques were justified.

***Multiple Imputation Procedure***

To address missing data and reduce bias from complete case analysis, Multiple Imputation by Chained Equations (MICE) was employed. MICE models the joint distribution of variables via a sequence of conditional models. For p variables with missing data, it estimates:


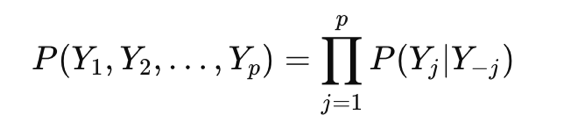


where each Yj is modeled conditional on all other variables Y−j.

We generated m=20 imputed datasets using predictive models incorporating all relevant covariates, treatment indicators, mediator variables, and outcomes to preserve relationships and uncertainty.

**Pooling of Estimates**

Statistical analyses (propensity score estimation, IPTW, regression, mediation) were run separately on each imputed dataset, yielding estimates Qi and variances Ui for i=1,…,m.

Final combined estimate Q and total variance T were calculated via Rubin’s rules:


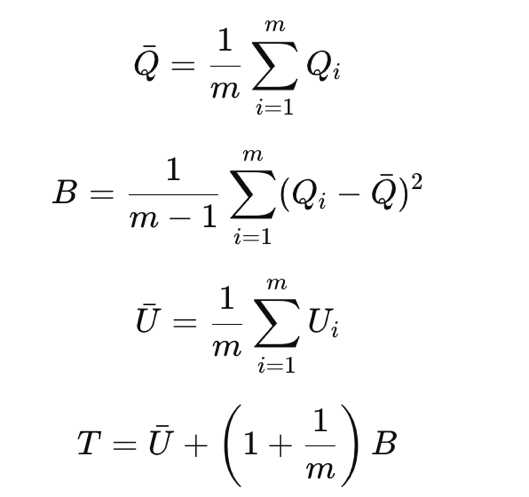


where B is the between-imputation variance, U the within-imputation variance, and T the total variance.

**Sensitivity Analyses**

To verify the robustness of results, sensitivity analyses compared outcomes from imputed datasets with complete case analyses, confirming consistency in effect estimates and statistical inference.

***Propensity Score***

*Definition and Estimation*

The **propensity score** e(X) is defined as the conditional probability of receiving the treatment (here, double lumen cannula, DL given a set of covariates X:


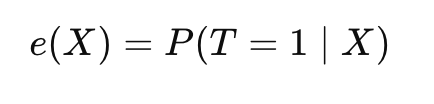


where T is the treatment indicator variable:


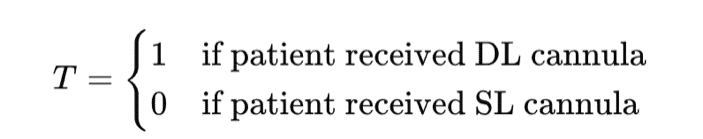


he propensity score was estimated using a multivariable logistic regression model:


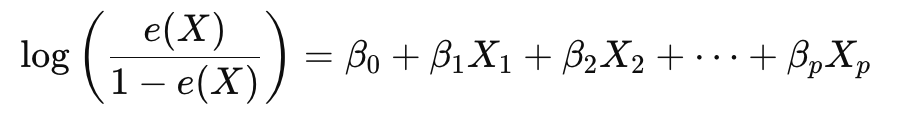


where X_1_,X_2_,…,X_p_ represent baseline clinical covariates including demographic, clinical parameters, treatment center, and treatment year.

*Covariate Balance Assessment*

To assess the effectiveness of the propensity score in balancing covariates between DL and SL groups, the Standardized Mean Difference (SMD) was calculated for each covariate:


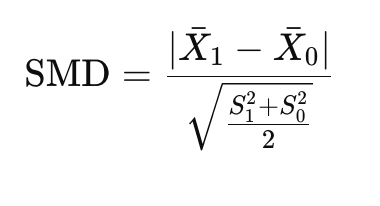


-
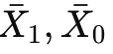
 are the means of the covariate in treated (DL) and control (SL) groups, respectively;
-
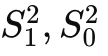
are the corresponding variances

An SMD<0.2 was considered indicative of adequate covariate balance, consistent with established recommendations (Austin, 2009). Balance diagnostics were performed both before and after the application of Inverse Probability of Treatment Weighting (IPTW), showing a substantial improvement in covariate balance post-weighting.

*Inverse Probability of Treatment Weighting (IPTW)*

IPTW weights each subject by the inverse of the probability of receiving the treatment they actually received:


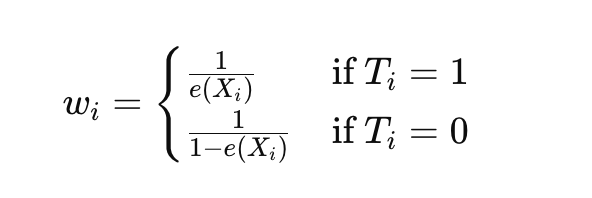


These weights create a pseudo-population in which the distribution of measured baseline covariates is independent of treatment assignment, approximating a randomized controlled trial.

*Model Calibration and Discrimination*

Model calibration was evaluated by calibration plots comparing predicted versus observed probabilities.

Discriminative ability was assessed by the **Area Under the Receiver Operating Characteristic Curve (AUC)**, with values closer to 1 indicating excellent discrimination.

*Clustering and Center-Level Adjustment*

Given the multicenter nature of the study and temporal variation, mixed-effects logistic regression models with random intercepts for center and treatment year were fitted:


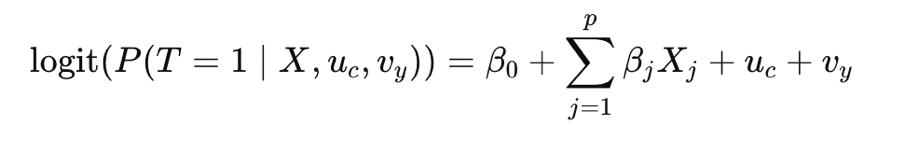


where:

-
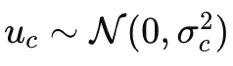
represents the random effect for center c;
- ​
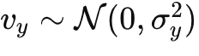
represents the random effect for year yyy.

This approach accounts for heterogeneity at the center and temporal levels, improving estimate validity.

***Univariable Analysis Using IPTW***

Univariable associations between treatment exposure (DL vs SL cannula) and outcomes were initially assessed using inverse probability of treatment weighting (IPTW) to control for confounding.

For each outcome YYY, we fitted a weighted regression model including only the treatment indicator TTT:

- For binary outcomes:


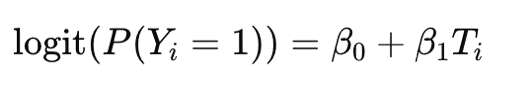


- For continuous outcomes:


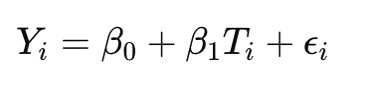


Observations were weighted by stabilized IPTW weights wiw_iwi​ derived from the propensity score model described previously, to create a pseudo-population balancing baseline covariates.

The estimated coefficient β1\beta_1β1​ represents the marginal effect of treatment on the outcome, adjusted for measured confounders through weighting.

Statistical inference was performed using robust sandwich variance estimators to account for weighting and potential clustering by clinical center.

***Multivariable Analysis Using IPTW***

To estimate the direct effect of treatment (DL vs SL cannula) on outcomes while adjusting for additional covariates and potential confounders, multivariable regression models were applied to the IPTW-weighted sample.

For each outcome YYY, the following weighted generalized linear model was specified:

- For binary outcomes (e.g., mortality):


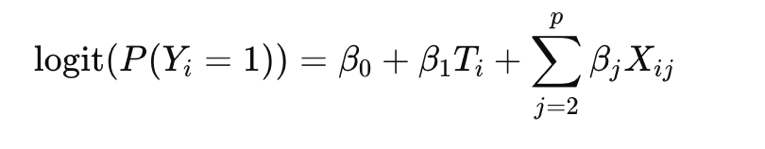


- For continuous outcomes:


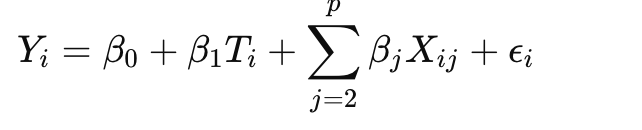


Where:

- T_i_ is the treatment indicator for patient i (1 for DL, 0 for SL).
- X_ij_ are covariates including center indicators, year of treatment, and clinically relevant confounders.
- β1 ​ estimates the adjusted effect of treatment.
- ϵ_i_ is the error term assumed to have mean zero.

The models were weighted by stabilized inverse probability of treatment weights wiw_iwi​ calculated from the propensity score, to achieve covariate balance and reduce bias.

Robust sandwich variance estimators were employed to obtain valid standard errors accounting for weighting and clustering at the center level.

Adjusted odds ratios (ORs), 95% confidence intervals (CIs), and p-values were derived from the model coefficients and their standard errors to assess statistical significance.

*Adjustment for Center and Year Effects*

Given heterogeneity in clinical practices and temporal changes, models included:

- Fixed effects γc\gamma_cγc​ for each clinical center ccc;
- Linear terms δ×Yeari\delta \times \text{Year}_iδ×Yeari​ for treatment year.

Thus, the logistic model extended to:


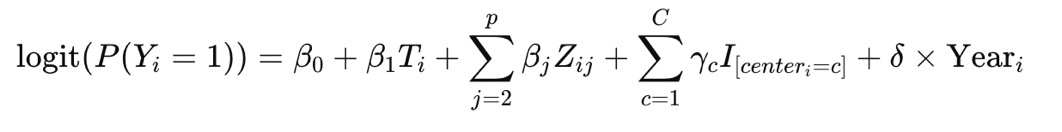


where I_[centeri=c]_ is an indicator function for center membership.

***Model Robustness and Sensitivity Analyses***

Robustness of the findings was assessed through several complementary approaches. Results obtained from the IPTW analysis were compared with those derived from propensity score matching and unweighted regression models to evaluate consistency. Sensitivity analyses were performed by excluding influential centers and observations with extreme weights to examine their impact on effect estimates. Alternative model specifications, including different covariate sets and weight trimming procedures, were also tested to assess the stability of the results.

For analyses involving multiple imputed datasets (mmm imputations), effect estimates θ^m\hat{\theta}_mθ^m​ and their variances V^m\hat{V}_mV^m​ were combined using Rubin’s rules as follows:

1. Pooled estimate:


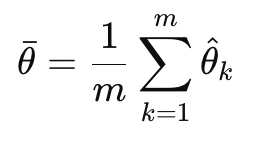


1. Within-imputation variance:


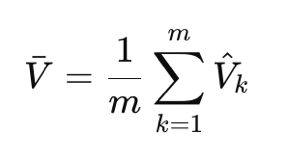


1. Between-imputation variance:


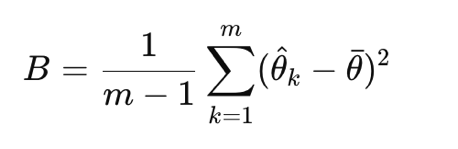


1. Total variance:


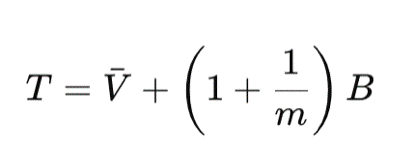


The standard error of the pooled estimate is then:


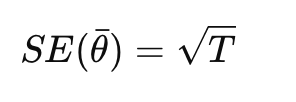


Confidence intervals and p-values were derived using these pooled estimates and variances.

***Interaction Analysis***

*Concept of Effect Modification*

Effect modification occurs when the effect of an exposure (here, double lumen cannula, DL) on an outcome varies depending on the level of another variable, termed the effect modifier. Identifying such interactions is critical for understanding heterogeneity in treatment effects and for tailoring clinical interventions.

*Methodology for Testing Interactions*

To evaluate potential effect modification, interaction terms between treatment status TTT (DL vs SL) and selected clinical covariates ZZZ (e.g., pre-cannulation arterial oxygen tension PaO2\text{PaO}_2PaO2​, serum creatinine) were incorporated into weighted regression models adjusted via IPTW:


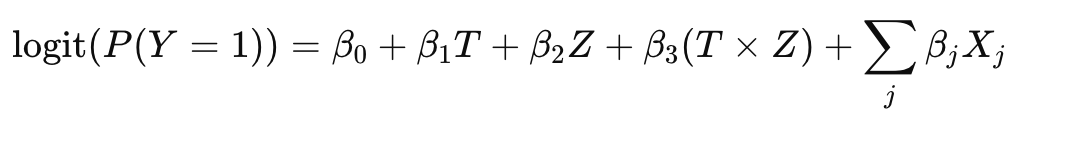


where:

- Y is the binary outcome;
- T is the treatment indicator (DL = 1, SL = 0);
- Z is the potential effect modifier;
- T×Z represents the interaction term;
- Xj are additional covariates.

Significance of the interaction term β_3_ ​ was assessed using Wald tests with robust standard errors, considering p<0.05 as statistically significant.

*Interpretation of Significant Interactions*

A statistically significant interaction indicates that the effect of DL cannulation on the outcome varies according to the level of the modifier variable. For example, a significant interaction between DL and arterial oxygen tension (PaO_2_) with p<0.01 suggests that the protective effect of DL cannulation is not uniform but depends on PaO_2_.

In our analysis, the benefit of DL cannulation was more pronounced among patients with lower PaO_2_ (i.e., hypoxemic patients), indicating that those with more severe hypoxemia derive greater survival advantage from the double lumen approach. Cannulation site (central vs peripheral) was likewise tested as an interaction term with cannula type (SL vs DL). Consistent with the main analysis, cannulation site was not associated with outcomes and did not significantly modify the comparative effect of SL vs DL (all P for interaction >0.05). Likely, oxygenator use at cannulation was also evaluated as a covariate and as an interaction term with cannula type. In keeping with the main results, oxygenator status was not independently associated with mortality or complications, and no significant interaction with SL vs DL was observed (all P for interaction >0.05). This supports the interpretation that baseline hypoxemia (PaO₂) is the clinically relevant effect modifier, rather than the presence of an oxygenator per se.

This finding provides important clinical insight, as it identifies a subgroup likely to benefit most from DL cannulation, thereby supporting more personalized treatment decisions.

***Causal Mediation Analysis***

*Basic Concepts: Direct and Indirect Effects*

Causal mediation analysis decomposes the total effect of an exposure (double lumen cannulation, DL) on an outcome into:

- The Average Direct Effect (ADE): the effect of DL on the outcome not mediated by intermediate variables;
- The Average Causal Mediation Effect (ACME): the effect mediated through one or more intermediate variables (mediators).

The proportion mediated quantifies the share of the total effect explained by the mediators.

*Multivariable Causal Mediation Models with Multiple Mediators*

We employed multivariate mediation models incorporating multiple postoperative biomarker mediators simultaneously. These models estimate both direct and indirect effects adjusted for baseline confounders.

*Calculation and Interpretation of ACME, ADE, and Proportion Mediated*

Using counterfactual frameworks, we estimated:


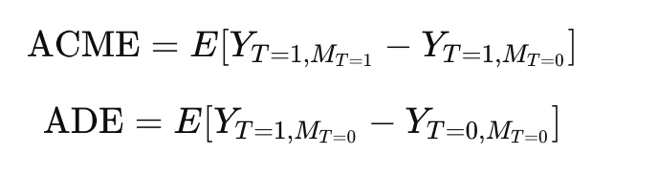


where Y is the outcome, T the treatment, and M the mediator.

Proportion mediated is computed as:


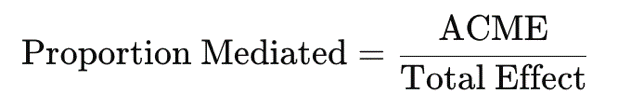


*Use of IPTW for Confounding Adjustment*

To control for confounding in both the treatment–mediator and mediator–outcome relationships, IPTW weights derived from propensity scores were incorporated into the mediation models.

*Assumptions for Valid Mediation Analysis*

Our analyses assume:

- No unmeasured confounding of the treatment–outcome, treatment–mediator, and mediator–outcome relationships;
- No mediator–outcome confounding affected by treatment (sequential ignorability);
- Correct model specification.

***Structural Equation Modeling (SEM)***

Structural Equation Modeling (SEM) was utilized to simultaneously estimate complex relationships among observed and latent variables, including direct and indirect pathways between double lumen (DL) cannulation, postoperative mediators, and clinical outcomes.

*Model Specification*

SEM allows the integration of multiple regression equations into a single coherent framework:


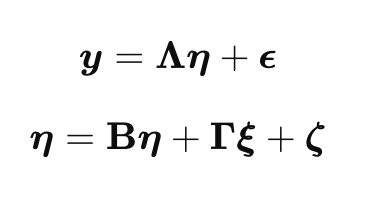


Where:

- **y** represents observed variables (e.g., biomarkers, outcomes);
- **η** are latent endogenous variables;
- **ξ** are latent exogenous variables (e.g., treatment assignment);
- **Λ** is the factor loading matrix;
- **B** represents relationships among endogenous latent variables;
- **Γ** relates exogenous to endogenous latent variables;
- **ζ** are measurement and structural error terms, respectively.

*Estimation and Fit Assessment*

Model parameters were estimated using maximum likelihood estimation with robust standard errors. Model fit was assessed using standard indices:

- Comparative Fit Index (CFI);
- Tucker-Lewis Index (TLI);
- Root Mean Square Error of Approximation (RMSEA);
- Standardized Root Mean Square Residual (SRMR).

*Interpretation*

SEM enables the quantification of complex mediation and moderation effects within a unified model, capturing the interdependence among multiple biomarkers and clinical outcomes. This approach provides deeper insight into the mechanistic pathways through which DL cannulation influences patient prognosis.

**Clustering Analysis**

*K-Means Clustering with Postoperative Biomarkers*

Unsupervised K-means clustering was applied to standardized postoperative biomarker data to identify clinically meaningful patient phenotypes.

*Clinical Characteristics and Outcomes of Clusters*

Clusters were characterized by distinct biomarker profiles, with differential clinical features and outcome rates, suggesting heterogeneity in pathophysiology and response to treatment.

*Clinical Relevance of Clustering*

Identifying these phenotypes aids in understanding disease mechanisms and may guide personalized therapeutic strategies.

**Model Validation**

**Predictive Performance Metrics**

Model discrimination was assessed via Receiver Operating Characteristic (ROC) curves and corresponding Area Under the Curve (AUC).

Calibration was evaluated using:

- Brier score;
- Calibration plots comparing predicted versus observed probabilities.

Supplementary Figures S9 and S10 depict these validation results.

**Final Manuscript Summary and Novel Contributions**

This study represents a comprehensive and methodologically rigorous evaluation of the impact of double lumen (DL) versus single lumen (SL) cannulation in a large multicenter observational cohort. Key novel aspects include:

- **Sophisticated Confounding Control:** Application of advanced propensity score methodologies including IPTW and multiple imputation to robustly adjust for baseline and treatment-related confounders, incorporating center- and year-level adjustments.
- **Innovative Causal Mediation Analysis:** Integration of multivariable causal mediation models assessing multiple postoperative biomarker mediators, quantifying direct and indirect pathways through which DL cannulation influences clinical outcomes.
- **Robust Interaction Testing:** Systematic evaluation of effect modification, identifying clinically relevant interactions such as between DL cannulation and pre-cannulation arterial oxygen tension (PaO₂), refining subgroup treatment benefit insights.
- **Unsupervised Phenotyping:** Identification of distinct patient clusters via K-means clustering based on postoperative biomarkers, revealing heterogeneous biological profiles linked to differential outcomes.
- **Comprehensive Model Validation:** Thorough model calibration and discrimination analyses, including ROC curves and Brier scores, affirming predictive reliability.

Collectively, these findings advance understanding of DL cannulation's mechanistic and clinical effects, provide a framework for personalized patient management, and establish methodological precedents for future cardiovascular ECMO research.

**Figure S1.** **Distribution of In-Hospital and 30-day Mortality by Cannula Type.** Bar plots showing the proportion of patients experiencing (A) in-hospital mortality and (B) 30-day mortality, stratified by cannulation strategy. Patients receiving **single-lumen (SL)** and **dual-lumen (DL)** cannulas are compared. Mortality rates were similar between groups for both outcomes, with slightly higher in-hospital mortality observed in the DL group. Values represent percentages over total group size. No statistical test is shown in this descriptive panel. This figure visually highlights differences between DL and SL groups to support the tabular results presented in Table S3.

**Figure S2.** Distribution of secondary outcomes stratified by cannula type (Double Lumen [DL] vs Single Lumen [SL]). Panels (A-C): proportion of patients with bleeding (A), continuous renal replacement therapy requirement (CRRT, B), and thromboembolism (C).
Panels (D-F): boxplots. The box represents the interquartile range (25th–75th percentile); the horizontal line indicates the median; the whiskers represent the minimum and maximum non-outlier values; individual dots represent outliers. The figure shows: (D) the composite outcome, defined as presence of bleeding and/or thromboembolism (Composite Outcome = Bleeding + Thromboembolism), (E) ICU length of stay, and (F) hospital length of stay, all stratified by cannula type.

The composite outcome (D) indicates the presence of at least one of the clinical events of interest (bleeding or thromboembolism). This figure visually highlights differences between DL and SL groups to support the tabular results presented in Table S3.

**Figure S3.** Baseline (pre-implant) distributions of key biomarkers stratified by cannula type (Double Lumen [DL] vs Single Lumen [SL]). Panels (A-F) show boxplots for lactate (Lactate), hemoglobin (HB), white blood cell count (WBC), serum creatinine (Creatinine), total bilirubin (Bilirubin), and aspartate aminotransferase (AST) measured before device implantation. These plots illustrate baseline differences between DL and SL groups.

**Figure S4.** Post-implant changes in key biomarkers stratified by cannula type (Double Lumen [DL] vs Single Lumen [SL]). Panels (A-F) show boxplots for the same biomarkers as in Figure S2, measured after device implantation. These plots depict post-implant status and allow comparison of biochemical responses between the two cannula types. Abbreviations: DL = Double Lumen; SL = Single Lumen; HB = Hemoglobin; WBC = White Blood Cell count; AST = Aspartate Aminotransferase.

**Supplementary Figure S5.**
Propensity score distribution before and after inverse probability of treatment weighting (IPTW), stratified by cannula type. The figure shows the distribution of estimated propensity scores for patients treated with dual-lumen (DL) and single-lumen (SL) cannulas, before and after IPTW. Substantial overlap between groups following weighting indicates effective adjustment for baseline covariates.An ASMD below 0.2 was considered indicative of adequate covariate balance.

**Supplementary Figure S6.** Stabilized inverse probability of treatment weights (IPTW), stratified by cannula type. Panel A is a boxplot. The box represents the interquartile range (25th–75th percentile); the horizontal line indicates the median; whiskers denote minimum and maximum non-outlier values; dots represent outliers. It shows the distribution of stabilized IPTW values by cannula group (single-lumen vs dual-lumen) using boxplots. Panel B displays the corresponding density plots. The distribution is centered around 1, with minimal extreme weights, indicating good weight stability and robust model performance.

**Supplementary Figure S7. Effective Sample Size (ESS) Distribution After IPTW Weighting**
Distributions of the effective sample size (ESS) for patients treated with single-lumen (SL, panel A) and dual-lumen (DL, panel B) cannulas, computed through 1,000 bootstrap replicates after inverse probability of treatment weighting (IPTW). Vertical dashed lines represent mean ESS for each group (SL = 63.6; DL = 115.3). Higher ESS values indicate greater statistical efficiency and confirm adequate overlap and weighting performance in both treatment arms. Abbreviations: ESS = Effective Sample Size; DL = Dual-lumen; SL = Single-lumen; IPTW = Inverse Probability of Treatment Weighting.

**Supplementary Figure S8. Temporal Trends in Cannulation Strategy and Early Mortality**
**Panel A**: Annual number of interventions stratified by cannulation type (DL vs SL). Panel B: Cannula use by year and center combination. Panel C: Early mortality trend over time by cannula type.
This figure illustrates evolving practice patterns and outcome variability across time, highlighting potential sources of bias.

**Supplementary Figure S9. Discrimination and Calibration for Predictive Model of Mortality**Panel A (top left): ROC curve for in-hospital mortality showing good discrimination (AUC = 0.76).
Panel B (bottom left): Calibration plot indicating good agreement between predicted and observed mortality probabilities (Brier = 0.178). Panel C (top right): ROC curve for early mortality (in-hospital or 30-day), showing good discrimination (AUC = 0.76). Panel D (bottom right): Calibration plot indicating good agreement between predicted and observed mortality probabilities (Brier = 0.194).

**Supplementary Figure S10 Predictive Models of Secondary Outcomes**Panel A (top left): ROC curve for bleeding showing good discrimination (AUC = 0.81).
Panel B (bottom left): Calibration plot indicating good agreement between predicted and observed probabilities (Brier = 0.115). Panel C (top right): ROC curve for CRRT showing excellent discrimination (AUC = 0.88). Panel D (bottom right): Calibration plot indicating good agreement between predicted and observed probabilities (Brier = 0.114).

**Supplementary Figure S11. Interaction effects between DL cannulation and baseline physiological variables on clinical outcomes.**

This figure illustrates significant statistical interactions between dual-lumen (DL) cannulation and baseline patient characteristics across selected outcomes, derived from IPTW-weighted logistic regression models.

Each panel shows an interaction plot where curves represent the adjusted probability or odds of the outcome stratified by cannulation type (DL vs SL) across values of a moderator variable. The intersection or divergence of curves indicates a statistically significant interaction. For completeness, oxygenator was included as a binary covariate, but curves are not shown due to lack of effect modification.

**Panels A (top right) B (top left) :** DL cannulation is associated with reduced in-hospital and 30-day mortality only in patients with lower PaO**₂**. The protective effect of DL decreases as pre-ECMO PaO₂ increases (interaction *p* < 0.01). Panel C: For CRRT requirement, DL confers a marked reduction in risk among patients with baseline creatinine ≥1.5 mg/dL, but not in those with preserved renal function (*p* = 0.012). Panel D: No significant interaction was found between DL and thrombocytopenia (platelets <150) in predicting bleeding risk (*p* = 0.73). No significant interaction was found between DL and oxygenator use in predicting mortality or complications (all P for interaction >0.05). Moreover, no significant interaction was found between DL and cannulation site (central vs peripheral) across outcomes (all P for interaction >0.05). For clarity, plots are not shown.

Abbreviations: DL = dual-lumen; SL = single-lumen; PaO₂ = arterial oxygen pressure; CRRT = continuous renal replacement therapy.

**Supplementary Figure S12. Multivariable causal mediation network for 30-day mortality.**

Directed acyclic graph (DAG) illustrating the multivariable causal mediation model evaluating the effect of dual-lumen (DL) cannulation on 30-day mortality via multiple postoperative biomarkers.

Each arrow represents a weighted, standardized path estimate (adjusted using inverse probability of treatment weighting). Green arrows indicate positive associations; red arrows indicate negative ones. Solid lines denote statistically significant effects (*p* < 0.05); dashed lines represent non-significant paths. Edge thickness is proportional to effect size.

This model integrates the joint mediating influence of lactate, creatinine, bilirubin, hemoglobin, and additional biochemical parameters, accounting for direct and indirect effects.

Compared to Figure 4, which quantifies the proportion of the total effect mediated by individual biomarkers, this figure maps the causal structure and interrelations between DL cannulation, intermediate physiological states (e.g., tissue hypoperfusion, renal function), and outcome. The strongest mediating pathways emerge through postoperative lactate and creatinine, reflecting their clinical importance as indicators of tissue perfusion and renal dysfunction. This systems-level perspective emphasizes that the beneficial effect of DL cannulation on survival is not direct but rather propagated through modulation of early organ function.

cannulation; CRR = CRRT requirement; TE = thromboembolism; Bld = bleeding.

**Supplementary Figure S13. Causal mediation diagram for bleeding and thromboembolic events.**
Directed acyclic graph (DAG) illustrating hypothesized mediation pathways linking dual-lumen (DL) cannulation to bleeding and thromboembolic complications.
Green arrows represent statistically significant causal paths (p < 0.05), while dashed grey arrows indicate non-significant associations. The model incorporates postoperative platelet count (PO2), creatinine (Crt), lactate (Lct), bilirubin (BIT), and hemoglobin (HBp) as plausible mediators of the effect of DL cannulation on clinical outcomes. The circles labeled "st_" and "dn_" represent latent variables for systemic inflammation and disease severity, respectively. Thickness of the arrows corresponds to the strength of the effect sizes. This figure complements the forest plot (Figure 4) by explicitly mapping the directional structure and potential mechanisms underlying bleeding and thromboembolic outcomes**. Abbreviations:** DL = dual-lumen cannulation; PO2 = platelet count; Crt = creatinine; Lct = lactate; BIT = bilirubin; HBp = hemoglobin; st_ = systemic inflammation latent variable; dn_ = disease severity latent variable.

**Supplementary Figure S14. Interaction between platelet count and cannulation type on bleeding risk.**

Interaction plot showing the predicted probability of bleeding across varying levels of baseline platelet count, stratified by cannulation type (DL vs SL). Both curves were estimated from weighted logistic regression models. Although the interaction between DL and platelet count was not statistically significant (*p* = 0.73), the figure illustrates the clinical hypothesis tested: In patients with platelet counts <150,000/μL, the difference in bleeding risk between DL and SL cannulation is minimal. In patients with higher platelet counts, bleeding risk decreases overall, but the DL-SL gap remains modest.

This plot is included for transparency and interpretability, despite the lack of a significant effect modification. Abbreviations: DL = dual-lumen; SL = single-lumen; Platelets = baseline platelet count (×10³/μL).

**Supplementary Figure S15. Structural equation model linking DL cannulation, organ dysfunction, and mortality.**

Heatmap showing the average values of key postoperative biomarkers and clinical outcomes across three patient clusters derived from unsupervised k-means analysis. Each row represents a variable; each column corresponds to a cluster. Cluster 1 (low-risk) shows favorable biochemical profiles and outcomes; Cluster 3 (high-risk) is associated with elevated lactate and creatinine, low hemoglobin, and higher rates of mortality and complications. This phenotypic stratification highlights early postoperative profiles with prognostic value following DL support. Abbreviations: CRRT = continuous renal replacement therapy.
